# Using museum specimens to estimate broad-scale species richness: Exploring the performance of individual-based and spatially explicit rarefaction

**DOI:** 10.1371/journal.pone.0204484

**Published:** 2018-10-31

**Authors:** Oyomoare L. Osazuwa-Peters, W. D. Stevens, Iván Jiménez

**Affiliations:** 1 Center for Conservation and Sustainable Development, Missouri Botanical Garden, St. Louis, MO United States of America; 2 Science and Conservation Division, Missouri Botanical Garden, St. Louis, MO United States of America; Università di Pisa, ITALY

## Abstract

Estimates of spatial patterns of broad-scale species richness are central to major questions in ecology, evolution and conservation. Yet, they are scarce due to incomplete information on species distributions. Often the only germane data derives from museum specimens collected during non-standardized sampling. Rarefaction, a promising approach to estimate broad-scale richness with these data, estimates the expected number of species represented in subsets of *n* specimens drawn from *N* specimens collected in a sampling unit. One version of rarefaction, known as individual-based rarefaction, assumes that the *N* specimens collected in a sampling unit constitute a random sample of individuals in that sampling unit. Another version, known as spatially explicit rarefaction, assumes that the *N* specimens collected in a sampling unit are spatially aggregated. We examined the working hypothesis that, when applied to museum specimen data, spatially explicit rarefaction is less biased than individual-based rarefaction because it reduces overestimation due to spatially aggregated sampling. We derived five predictions from this working hypothesis and tested them using computer simulation experiments based on a database of 129,782 plant specimens from Nicaragua, and sampling units of 5 x 5, 50 x 50, and 100 x 100 km. One experiment was a negative control, whereby we simulated collection of randomly chosen individuals from each sampling unit. In contrast, three other experiments included spatially aggregated sampling. In all experiments we applied individual-based and spatially explicit rarefaction to estimate richness, with *n* = 200 and *n* = 500 specimens. As expected, the experiment designed as a negative control did not support the working hypothesis. The other three experiments supported the working hypothesis in analyses of larger sampling units, but not in 5 x 5 km sampling units. The predictions we derived from the working hypothesis can be used to assess which rarefaction version is best in particular systems.

## Introduction

**Fig 1:**
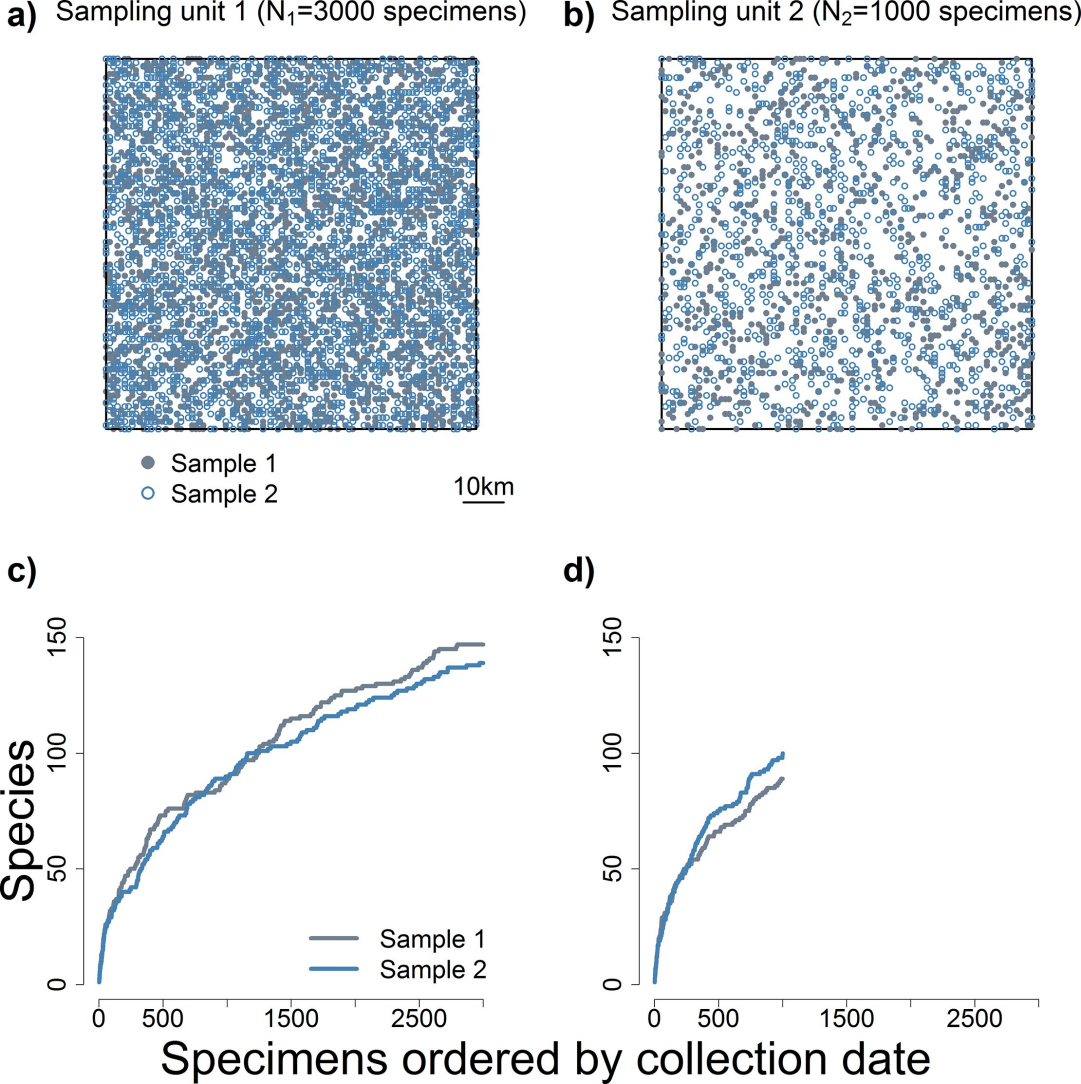
Differences in sampling effort confound comparisons of species richness between sampling units. Panels (a) and (b) show maps derived from a simulation of two 100 x 100 km sampling units, and the collection localities of specimens sampled from them. Both sampling units were simulated to have identical total number of species, species abundance distribution and geographic distribution of species. Details of the simulation are described in Methods. In panel (a), blue open symbols show collection localities for one set of specimens, and grey closed symbols for a second set of specimens. Both sets contain *N*_*1*_ = 3000 specimens, each constituting a random sample of individuals from the sampling unit. Therefore, the two sets of specimens differ in terms of the individuals and species included. Accordingly, the two sets of specimens produce different accumulation curves, shown in panel (c). Accumulation curves display the total number of species revealed as additional specimens are added to the pool of all previously collected specimens [11]. The differences between accumulation curves in panel (c) illustrate how sampling variation in accumulation curves derives from variation in the composition of samples (i.e., sets of specimens) in terms of individuals and species. Panels (b) and (d) show respective information for the second 100 x 100 km sampling unit, with two sets of *N*_*1*_ = 1000 specimens. Comparison of the number of species known to occur in each sampling unit would be invalid, because it would reflect differences in sampling effort and not differences in species richness.

Understanding spatial patterns of broad-scale species richness is a major goal in ecology and evolutionary biology [1,2], with implications for biodiversity conservation [3,4]. Yet, quantifying broad-scale species richness is often difficult due to uncertainty about the geographic distribution of species. This issue, known as the “Wallacean shortfall” [5,6], is most severe for studies focusing on highly diverse taxa, including vascular plants and invertebrates [7–10]. Often the only germane information available derives from natural history museum specimens collected during non-standardized sampling. Obtaining from these data complete counts of the number of species occurring within relatively large areas is often virtually impossible. In these instances, taxon sampling curves that relate species richness in the ordinate to sampling effort in the abscissa [11], are unlikely to come near an asymptote. Therefore, raw counts of species occurring within large geographic areas would be highly dependent on sampling effort and thus not suitable estimates for studying patterns of broad-scale species richness. For example, it would be invalid to compare the number of species known to occur in two equal-area sampling units that differed in sampling effort (Fig 1).

To make valid comparisons of species richness across sampling units, sampling effort should ideally be standardized to a common number of specimens [12], hereafter *n* (e.g., *n* = *N*_*2*_ in Fig 1). Therefore, for sampling units that have samples larger than *n*, the challenge is to determine the number of species that would have been found if only *n* specimens were collected, *S*_*n*_. It might be tempting to address this challenge by determining the number of species represented in the first *n* specimens collected in a sampling unit, using species accumulation curves that record the number of species revealed through time as specimens are collected in a sampling unit (Fig 1, [11]. However, this solution would fall short because the number of species represented in *n* specimens varies with the order in which specimens are arranged, and there are no grounds to select any particular sequence of specimens, chronological or otherwise [13,14]. In other words, *S*_*n*_ exhibits sampling variation that stems from the order in which specimens are collected. Additionally, *S*_*n*_ exhibits sampling variation due to sample composition in terms of individuals and species (Fig 1).

**Fig 2:**
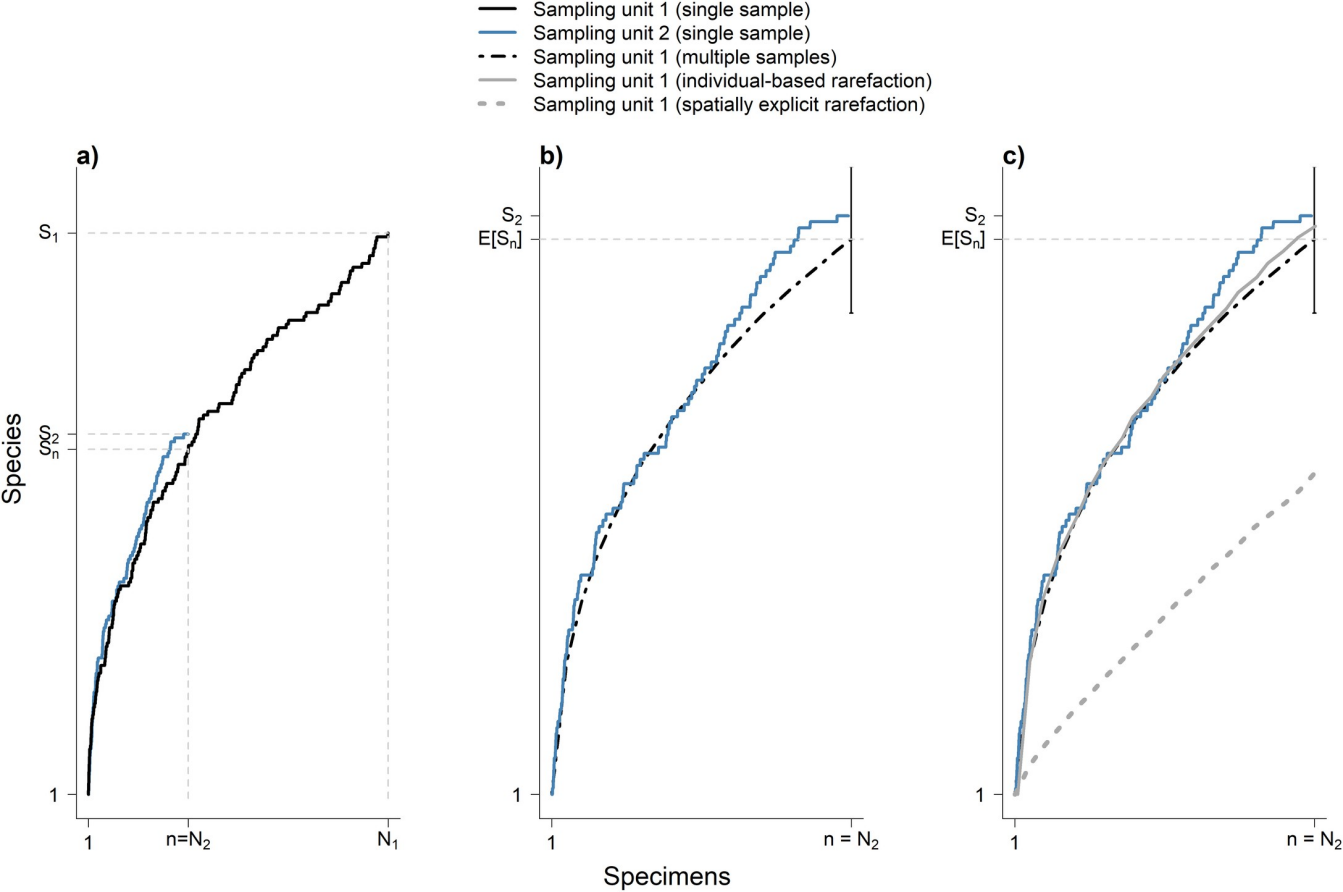
Individual-based rarefaction is ideal to control for differences in random sampling effort. Panel (a) shows species accumulation curves for the two simulated sampling units shown in Fig 1. They differ in sampling effort but are identical in total number of species, species abundance distribution and geographic distribution of species. The number of species found in sampling unit 1, *S*_*1*_, is higher than the number of species found in sampling unit 2, *S*_*2*_. This difference is only due to higher collection effort in the former sampling unit: *N*_*1*_ > *N*_*2*_. In fact, the number of species represented in the first *n = N*_*2*_ specimens collected in sampling unit 1, *S*_*n*_, is lower than *S*_*2*_; but this difference reflects sampling variation in species accumulation curves (Fig 1). Panel (b) describes sampling variation in the species accumulation curve for sampling unit 1. The error bar at *n* = *N*_*2*_ specimens represents a range that includes 95% (from 2.5–97.5 percentile) of species richness values at *n* = *N*_*2*_ for 100 sets of specimens drawn from sampling unit 1. Each of these sets of specimens constitutes a random sample of *n* = *N*_*2*_ individuals from sampling unit 1. For lower number of specimens (*n* < *N*_*2*_) only the average curve is shown. Note that the 100 accumulation curves for sampling unit 2 are true replicates: they are not generated by reordering a single set of specimens. At *n* = *N*_*2*_, the accumulation curve for sampling unit 2 falls well within the sampling variation for sampling unit 1, indicating no differences in species richness between the two sampling units. Panel (c) shows the individual-based rarefaction curve for sampling unit 1, generated by reordering 1000 times a single accumulation curve. The rarefaction curve falls well within the sampling variation for sampling unit 1. Indeed, individual-based rarefaction provides the minimum variance unbiased estimator of the expected number of species in a random sample of individuals from a sampling unit (see text). On the other hand, the curve showing spatially explicit rarefaction, generated from 1000 spatially explicit subsamples of the specimens in a single accumulation curve (see text), does not perform well in this simulation.

To account for sampling variation in *S*_*n*_, it would be useful to estimate the expected number of species represented in *n* specimens from a sampling unit, hereafter *E*[*S*_*n*_]. In principle, this may be accomplished by scaling taxon sampling curves using “individual-based rarefaction” [11,12]. Individual-based rarefaction is based on repeatedly and randomly drawing subsets of *n* specimens from the pool of all *N* specimens collected in a sampling unit, to calculate the average number of species represented in subsets of *n* specimens, hereafter *E*[*S*_*n*.*r*_]. There are formulas for estimating *E*[*S*_*n*.*r*_] and its unconditional variance [12,15]. If the pool of *N* specimens collected in a sampling unit is a random sample from the (potentially very large) set of individuals occurring in the sampling unit (Fig 1), then individual-based rarefaction would provide the minimum variance unbiased estimator of the expected number of species in a random sample of size *n* from the set of individuals in the sampling unit (Fig 2; [16]. Thus, *E*[*S*_*n*.*r*_] would be an estimator of *E*[*S*_*n*_] that allows comparison of species richness to other sampling units where sampling effort had been lower. Such comparisons would reflect the total number of species in the sampling units and the respective species abundance distributions [17,18], but not differences in sampling effort among sampling units.

Individual-based rarefaction has been used to analyze data from standardized surveys [18–20], where sampling designs may approach the scenario in which the pool of *N* specimens is a random sample of individuals occurring in the sampling unit (Figs 1 and 2). Individual-based rarefaction has also been applied to estimate broad-scale species richness based on data from museum specimens [8,21,22], but these latter data are unlikely to meet assumptions typically required to apply rarefaction [18]. In particular, the pool of *N* specimens collected in a sampling unit is unlikely to be a random sample of the individuals in that sampling unit, thus deviating from the sampling scenario described in Figs 1 and 2. By example, collection effort often shows positive spatial autocorrelation at relatively short distances and, therefore, it is commonly spatially aggregated within sampling units [23]. Additionally, collectors may typically try to maximize the number of species collected, generally making few vouchers of each species [24]. Furthermore, collectors may preferentially target certain groups of organisms [25], such as species from a particular taxon or ecological guild. Yet, recent work suggests that individual-based rarefaction may be the best currently available approach to scale taxon sampling curves derived from museum specimens [8,26], even if the assumptions of rarefaction are not strictly met and the resulting richness estimates are only approximate. Nonetheless, little is known about the performance of rarefaction in this context, and it would seem useful to explore the performance of different versions of rarefaction when applied to data from museum specimens.

**Fig 3:**
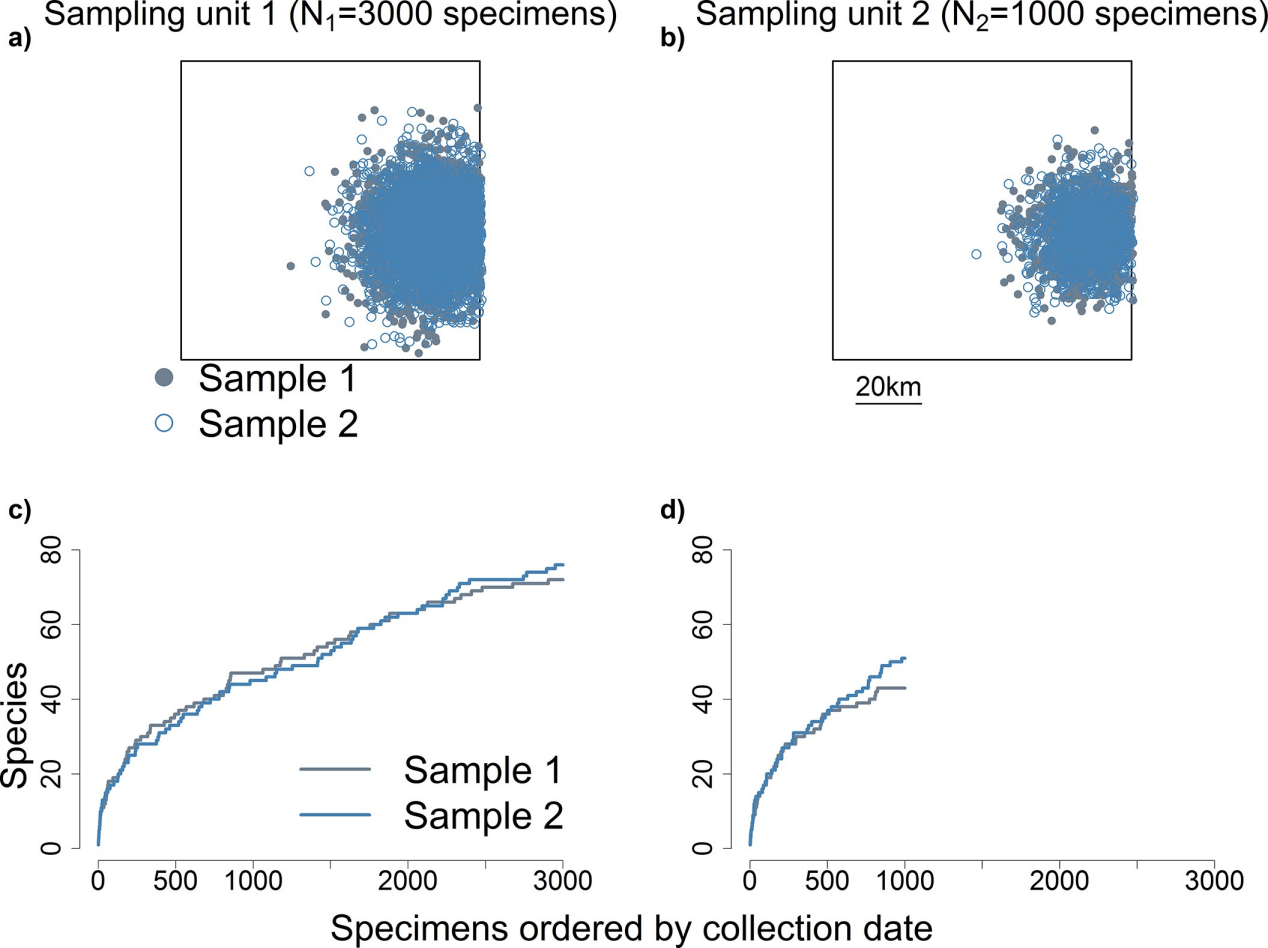
Differences in spatially aggregated sampling effort confound comparisons of species richness between sampling units. Panels (a) and (b) show maps derived from a simulation of two 100 x 100 km sampling units, and the collection localities of specimens sampled from them. Both sampling units were simulated to have identical total number of species, species abundance distribution and geographic distribution of species. Details of the simulation are described in Methods. In panel (a), blue open symbols show collection localities for one set of specimens, and grey closed symbols for a second set of specimens. Both sets contain *N*_*1*_ = 3000 specimens, each constituting a spatially aggregated sample of individuals from the sampling unit. Panel (b) shows respective information for a second sampling unit. The spatial aggregation of specimens in panels (a) and (b) was simulated using an “expanding” bivariate normal distribution, described in the Methods section. Panels (c) and (d) show accumulation curves for the sets of specimens in panels (a) and (b), respectively. The difference in sampling effort (*N*) between (a) and (b) confounds the comparison of accumulation curves in (c) and (d).

**Fig 4:**
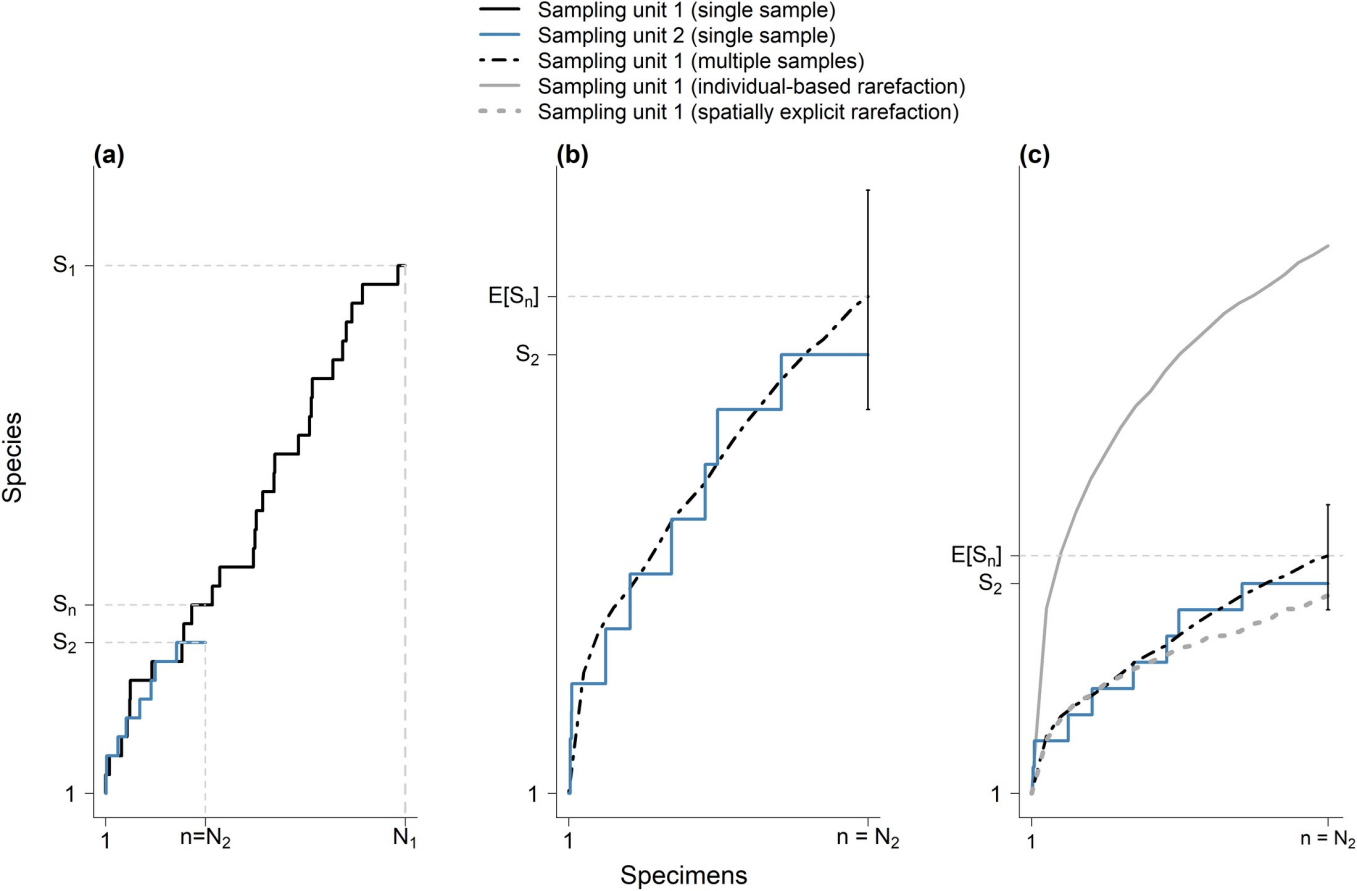
Spatially explicit rarefaction is thought to be useful to control for differences in aggregated sampling effort. Panel (a) shows species accumulation curves for the two simulated sampling units shown in Fig 3. These sampling units differ in sampling effort but are identical in total number of species, species abundance distribution and geographic distribution of species. Panel (b) describes sampling variation in the species accumulation curve for sampling unit 1. The error bar at *n* = *N*_*2*_ specimens represents a range that includes 95% (from 2.5–97.5 percentile) of species richness values at *n* = *N*_*2*_ for 100 sets of specimens drawn from sampling unit 1. Each of these sets of specimens constitutes a spatially explicit sample of *n* = *N*_*2*_ individuals from sampling unit 1. For lower number of specimens (*n* < *N*_*2*_) only the average curve is shown. Note that the 100 accumulation curves for sampling unit 2 are true replicates: they are not generated by reordering a single set of specimens. At *n* = *N*_*2*_, the accumulation curve for sampling unit 2 is within the sampling variation for sampling unit 1, indicating no differences in species richness between the two sampling units. Panel (c) shows the spatially explicit rarefaction curve for sampling unit 1, generated by reordering a single accumulation curve 1000 times. In each instance, spatially explicit reordering of specimens was based on spatial proximity of all other specimens from an initial randomly selected specimen. The spatially explicit rarefaction curve falls well within the sampling variation for sampling unit 1. On the other hand, the curve showing individual-based rarefaction, generated from 1000 random subsamples of the specimens in a single accumulation curve (see text), does not perform well in this simulation.

As a starting point, we here focus on only one source of the disparity between the sampling design assumed in individual-based rarefaction and the way museum specimens are actually collected in the field: the spatial aggregation of sampling activities or, in other words, positive spatial autocorrelation of sampling effort at relatively short distances. Sampling effort by collectors is typically heavily constrained by the spatial layout of access routes and points [8,27,28], and characterized by high concentration in a few sites [23,29]. This leads to aggregation of sampling effort within large sampling units (Fig 3). Additionally, species occurrences are also typically aggregated within large sampling units [30], showing positive spatial autocorrelation at relatively short distances, as documented by patterns of distance decay of biotic similarity [31,32]. This combination of spatial aggregation in sampling effort and in species occurrences introduces positive bias in *E*[*S*_*n*.*r*_] as an estimator of *E*[*S*_*n*_] [11,33]. Thus, application of individual-based rarefaction to museum specimen data likely results in overestimation of the number of species that would have been found with less sampling effort (Fig 4).

This positive bias in estimates of *E*[*S*_*n*_] might potentially be lessened by using another version of rarefaction known as “spatially explicit rarefaction” [34,35], (previously called “spatially-constrained rarefaction”). This rarefaction approach aims to make valid comparisons of species richness across sampling units by simultaneously controlling for the number of specimens (as in individual-based rarefaction) and for the spatial arrangement of sampling activities. It was originally developed for multi-individual samples (i.e., “sample-based rarefaction”, [11]), but it may be adapted to data from museum specimens. In particular, in spatially explicit rarefaction, the spatial proximity of the collecting localities of specimens is considered when drawing subsets of *n* specimens from the pool of all *N* specimens collected in a sampling unit. These subsets are obtained by selecting specimens whose collecting localities are adjacent to each other in geographic space, thus mimicking spatial aggregation in sampling activities. Importantly, adjacency of collecting localities may be defined in multiple ways [31]. For simplicity, here we adopt a definition of adjacency based on the k-nearest neighbor. Under this definition, spatially explicit subsets of *n* specimens are operationally obtained by first randomly choosing an initial specimen and subsequently adding the *n*-1 specimens with collecting localities closest (in geographic space) to that of the initial specimen. Then, the average number of species represented in these spatially explicit subsets of *n* specimens is calculated, hereafter *E*[*S*_*n*.*ser*_]. Judging by previous applications of spatially explicit rarefaction to standardized surveys [32–34], *E*[*S*_*n*.*ser*_] may show less upward bias than *E*[*S*_*n*.*r*_] as an estimator of *E*[*S*_*n*_] when sampling effort is spatially aggregated (Fig 4C). However, there seems to be no previous study examining the extent to which spatially explicit rarefaction, when applied to data from museum specimens collected during non-standardized sampling, outperforms individual-based rarefaction. Indeed, we are not aware of any previous application of spatially explicit rarefaction to data from museum specimens. Therefore, it seems useful to empirically examine whether spatially explicit rarefaction can improve estimation of *E*[*S*_*n*_] when applied to data from museum specimens.

Accordingly, in this study we examined the working hypothesis that, when estimating broad-scale species richness using data from museum specimens, spatially explicit rarefaction is less biased than individual-based rarefaction, because it reduces overestimation of *E*[*S*_*n*_] due to spatial aggregation of sampling activities. We developed and tested five predictions derived from this hypothesis. Importantly, all five predictions can be tested with data from museum specimens and, thus, used to explore the performance of individual-based and spatially explicit rarefaction in different study systems. Below we first describe the rationale used to derive these five predictions. Then, we present the test of the predictions based on computer simulations experiments, and on a comprehensive specimen database of Nicaraguan vascular plants.

### Predictions

All predictions were derived under the assumption that sampling units were large (≥ 25 km^2^) equal-area grid cells, sampled well-enough to be at least tentatively included in studies of broad-scale species richness. The predictions below do not apply, for example, to the extreme case in which all *N* specimens in a sampling unit come from a single locality.

Prediction 1 focuses on the variance across sampling units in the area covered by the collection localities of the subset of *n* specimens used to estimate *E*[*S*_*n*_]. By geographically restricting these subsets, spatially explicit rarefaction would be supposed to reduce differences across sampling units in the area covered by collection localities of the subsets of specimens used to estimate *E*[*S*_*n*_]. Hence, prediction 1 states that the variance across sampling units in the area covered by the collection localities of subsets of *n* specimens is lower for spatially explicit rarefaction than for individual-based rarefaction. Note that prediction 1 may fail to be supported even if collection effort is spatially aggregated, because sampling units may differ markedly in the pattern of aggregation of collecting localities, making it difficult to simultaneously control for the number of specimens and the spatial arrangement of sampling activities (S1A and S1C Appendices).

Prediction 2 compares two quantities obtained for each sampling unit. One of these quantities is the expected number of species in subsets of *n* specimens obtained by individual-based rarefaction, *E*[*S*_*n*.*r*_]. The other quantity is the number of species represented in the first *n* specimens of the accumulation curve, hereafter *S*_*n*.*a*_. Prediction 2 states that, on average across sampling units, *S*_*n*.*a*_ is smaller than *E*[*S*_*n*.*r*_]. This prediction derives from the fact that, when sampling designs approach the scenario in which the pool of *N* specimens collected from a given sampling unit is a random sample of the individuals occurring in the sampling unit (as described in Figs 1 and 2), individual-based rarefaction curves are statistical expectations of accumulation curves [11]. Therefore, in this scenario where the assumption of random sampling is satisfied, stochasticity in the chronological order in which specimens are collected would cause *S*_*n*.*a*_ to be larger than *E*[*S*_*n*.*r*_] in some sampling units, and smaller in others. However, on average across all sampling units, *S*_*n*.*a*_ would not be different from *E*[*S*_*n*.*r*_] in this scenario where the assumption of random sampling is satisfied. In contrast, if as proposed by the working hypothesis, individual-based rarefaction overestimates the number of species that would have been found with less effort due to spatial aggregation of sampling activities, then differences between *S*_*n*.*a*_ and *E*[*S*_*n*.*r*_] would not be attributable only to stochasticity in the chronological order in which specimens are collected. In this case, individual-based rarefaction curves would display a systematic tendency to lie above accumulation curves (Fig 4C, [11]). Thus, on average across sampling units, *S*_*n*.*a*_ would be smaller than *E*[*S*_*n*.*r*_].

Prediction 3 compares *E*[*S*_*n*.*r*_] to the expected number of species in subsets of *n* specimens obtained by spatially explicit rarefaction, *E*[*S*_*n*.*ser*_]. This prediction states that, on average across sampling units, *E*[*S*_*n*.*r*_] is larger than *E*[*S*_*n*.*ser*_] (Fig 4C). This prediction derives from the working hypothesis because, for any given sampling unit, a subset of *n* specimens obtained by individual-based rarefaction will (by definition) tend to include specimens from a wider area than a subset of *n* specimens obtained by spatially explicit rarefaction. Thus, according to the species-area relationship, *E*[*S*_*n*.*r*_] will tend to be larger than *E*[*S*_*n*.*ser*_].

Prediction 4 compares two absolute differences obtained for any sampling unit. The first is the absolute difference between *E*[*S*_*n*.*r*_] and *S*_*n*.*a*_, hereafter | *E*[*S*_*n*.*r*_] – *S*_*n*.*a*_ |. The second is the absolute difference between *E*[*S*_*n*.*ser*_] and *S*_*n*.*a*_, hereafter | *E*[*S*_*n*.*ser*_] – *S*_*n*.*a*_ |. These two differences measure absolute deviations between expected values of species richness obtained via rarefaction and the corresponding observed values obtained from accumulation curves. As stressed previously, valid rarefaction curves are statistical expectations of accumulation curves [11]. Therefore, if spatially explicit rarefaction is more accurate than individual-based rarefaction, as proposed by the working hypothesis, then | *E*[*S*_*n*.*r*_] – *S*_*n*.*a*_ | should be larger than | *E*[*S*_*n*.*ser*_] – *S*_*n*.*a*_ | (Fig 4C), on average across sampling units.

Prediction 5 focuses on the effect of sampling unit size on the magnitude of overestimation of *E*[*S*_*n*_] incurred by individual-based rarefaction. It states that as sampling unit size increases, individual-based rarefaction will more severely overestimate *E*[*S*_*n*_], because as sampling unit size increases the spatial aggregation of sampling activities and species occurrences becomes stronger, due to higher environmental heterogeneity across larger spatial extents. Thus, as sampling unit increases, the differences between quantities compared in predictions 2, 3, and 4 should increase.

## Methods

### Study system

We tested the five predictions above using computer simulation experiments based on the known flora of Nicaragua, which includes 5,982 species in 247 families of vascular plants. A comprehensive description of this flora has been constantly updated since it was first published [36], and it is thoroughly documented in the Tropicos database under the Flora de Nicaragua Project (http://www.tropicos.org/Project/FN), where records of geo-referenced and taxonomically verified specimens have been continuously curated by one of us (W. D. Stevens). This set of records contains the majority of specimens ever collected in Nicaragua, including historical collections now deposited in European herbaria. From this set of records, we excluded specimens without collection coordinates or date as well as specimens of cultivated plants, specimens not determined to species, or not yet accepted by the Flora de Nicaragua. After these exclusions, we had 129,782 specimen records representing 5,742 species (S2 Appendix).

We focused on examining the working hypothesis as applied to sampling units at three spatial scales: 5 x 5, 50 x 50, and 100 x 100 km. At each scale, we conducted two versions of the analyses, one for rarefaction based on subsets of *n* = 200 and the other for rarefaction based on subsets *n* = 500 specimens. The combination of three spatial scales and two *n* values defined six cases in each of the computer simulation experiments described next.

### Computer simulation experiments

We conducted four computer simulation experiments to examine the working hypothesis. In all experiments, we adopted a paired-test protocol in which two treatments, individual-based and spatially explicit rarefaction, were applied to all sampling units. The response variables were quantities addressed by the five predictions described above: area covered by subsets of *n* specimens (prediction 1), *E*[*S*_*n*.*r*_]- *S*_*n*.*a*_ (predictions 2 and 5), *E*[*S*_*n*.*r*_]- *E*[*S*_*n*.*ser*_] (predictions 3 and 5), | *E*[*S*_*n*.*r*_]−*S*_*n*.*a*_ | – | *E*[*S*_*n*.*ser*_] – *S*_*n*.*a*_ | (predictions 4 and 5).

As explained in detail below, different experiments preserved to different degrees four aspects of the data on specimens from Nicaragua (Table 1). The first aspect is the spatial pattern of plant richness across Nicaragua (Table 1). We consider this pattern to be an aspect of the data in the sense that it is the pattern sampled by the specimens from Nicaragua and, thus, determine properties of these data. The second and third aspects are largely self-explanatory: number of specimens within each sampling unit, and spatial distribution of the collecting localities of specimens within sampling units (Table 1). Operationally, we defined the distribution of collecting localities at a scale of 1 x 1 km grid cells within sampling units. The fourth aspect of the data on specimens from Nicaragua is species detectability, defined as the relationship between the probability of collecting a given species at a site (i.e., a 1 x 1 km grid cell) and the relative abundance of that species at the site ([37], Table 1).

**Table 1:** Four major aspects of the data on museum specimens from Nicaragua (second to fifth columns) that were simulated or preserved in four computer experiments (first column).

Experiment	Spatial pattern of plant richness across Nicaragua	Number of specimens within sampling units	Spatial distribution of the collecting localities of specimens within sampling units	Species detectability (*sensu* [37]
**Experiment A**	Simulated; continuum theory model	Preserved	Simulated; bivariate uniform distribution	Simulated; proportional to relative local abundance
**Experiment B**	Simulated; continuum theory model	Preserved	Simulated; “expanding” bivariate normal distribution	Simulated; proportional to relative local abundance
**Experiment C**	Simulated; continuum theory model	Preserved	Preserved	Simulated; proportional to relative local abundance
**Experiment D**	Preserved	Preserved	Preserved	Preserved

The first experiment, experiment A, served as a negative control because it rendered the working hypothesis false. Specifically, this experiment simulated a case in which individual-based rarefaction was ideal to control differences in sampling effort among sampling units, as depicted in Figs 1 and 2. In this case, sampling effort was uniformly distributed across geographic space within each unit (Fig 1), so valid comparisons of species richness among sampling units may be performed by controlling for the number of specimens using individual-based rarefaction (Fig 2), without worrying about the spatial arrangement of sampling activities. Indeed, attempting to apply spatially explicit rarefaction in this case would lead to under-estimation of species richness (Fig 2C).

**Fig 5:**
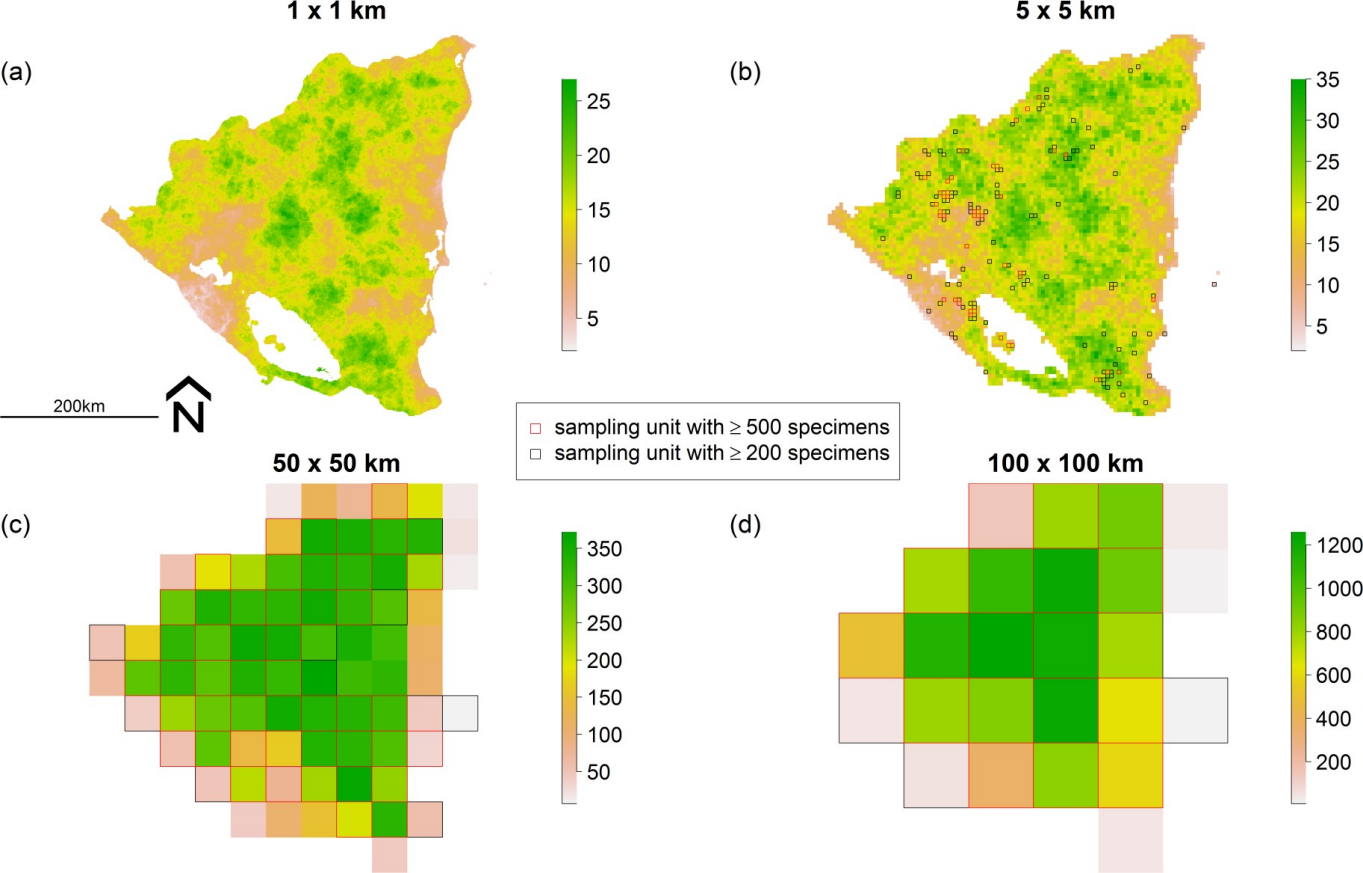
Simulated spatial pattern of plant richness based on a continuum theory model [38]. Spatial pattern of plant richness across Nicaragua shown for grid cells of (a) 1 x 1 km, (b) 5 x 5 km, (c) 50 x 50 km, and (d) 100 x 100 km. The color scales for each panel show total number of species occurring in grid cells. In panels (b), (c) and (d), black and red grid lines show sampling units with at least 200 and 500 specimens, respectively. See S9 Appendix for the distribution of specimens per sampling unit at spatial scales of 5 x 5, 50 x 50 and 100 x 100 km.

**Fig 6:**
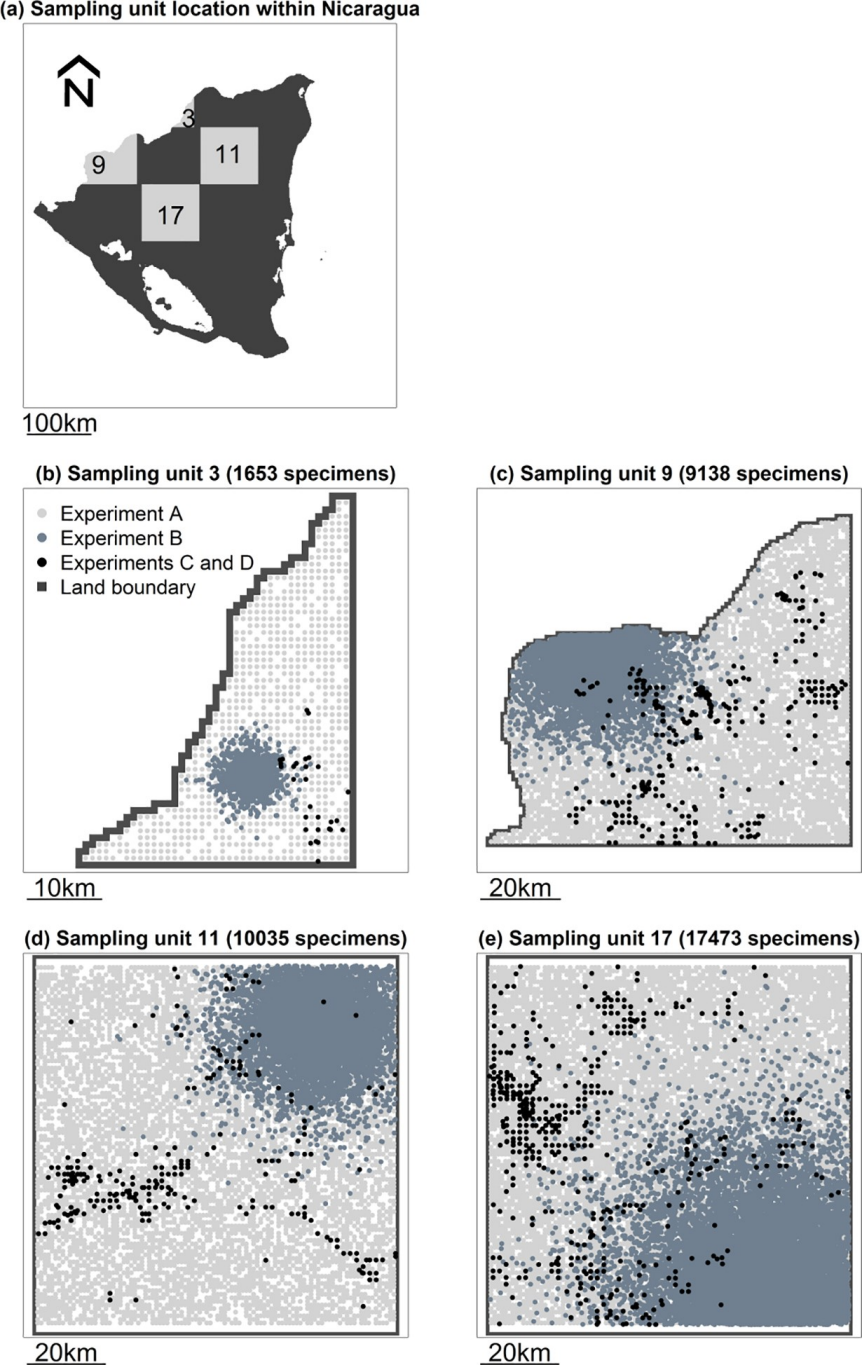
Spatial distribution of collecting localities of specimens within sampling units in computer simulation experiments. Four sampling units (100 x 100 km) were arbitrarily selected for illustration purposes. Experiments C and D have the same spatial distribution of collecting localities: the observed distribution of collecting localities of herbarium specimens from Nicaragua. Spatial scale bars are shown at the bottom of each panel.

Experiment A preserved only one aspect of the data on specimens from Nicaragua: the number of specimens within each sampling unit. The spatial pattern of richness across Nicaragua, the spatial distribution of collecting localities within sampling units, and species detectability were not preserved (Table 1). We simulated the spatial pattern of plant richness across Nicaragua (second column in Table 1) using a model based on continuum theory [30,38]. In particular, we simulated the abundance of 15,000 species across Nicaragua at a resolution of 1 x 1 km grid cells (see S3 Appendix for details, and S4 Appendix for R code and data). Therefore, we obtained the total number and identity of simulated species occurring at each 1 x 1 km grid cell across Nicaragua, and the respective species abundance distribution (SAD, Fig 5, S3 Appendix). To simulate the spatial distribution of the collecting localities within sampling units (third column in Table 1), we randomly drew coordinates from a bivariate uniform distribution across each sampling unit, at a resolution of 1 x 1 km (Fig 6). Once collecting localities were randomly assigned to particular 1 x 1 km grid cells, we assigned species to specimens according to species detectability (fourth column in Table 1), which equaled the relative abundance of species at the respective 1 x 1 km grid cell. Thus, the probability that a specimen represented a given species was proportional to the relative local abundance of that species at the collection locality.

Unlike experiment A, the next three experiments represent cases in which sampling activities were spatially aggregated. Thus, in contrast to experiment A, the design of the next three experiments did not render the working hypothesis false. Experiment B preserved only one aspect of the data on specimens from Nicaragua: the number of specimens within sampling units (Table 1). In this experiment, the spatial pattern of plant richness across Nicaragua and detectability were the same as in experiment A. But in contrast to experiment A, in experiment B we simulated an aggregated distribution of collecting localities within sampling units, using an “expanding” bivariate normal distribution (Fig 6, Table 1, S5 Appendix). To do so, we first ordered specimens chronologically, according to their collection date. Then, sequentially, we simulated the coordinates for their collecting localities by sampling from a bivariate normal distribution. The centroid of this distribution was randomly placed within the sampling unit. For the first specimen (with the oldest collection date), the respective variance-covariance matrix had covariance zero and variances (diagonal terms) equal to 500 m^2^. For every subsequent specimen, the variances increased by 1.5625 m^2^, thus expanding the original bivariate normal distribution. Whenever draws from these distributions resulted in coordinates outside the sampling unit, we drew additional values as needed to obtain coordinates inside the sampling unit. Thus, in experiment B, collecting localities within each sampling unit were aggregated around a randomly selected site (the centroid of the bivariate normal distributions), but were increasingly likely to be farther away from that site as more specimens were collected (Fig 6).

Experiment C preserved two aspects of the data on specimens from Nicaragua: the number of specimens within sampling units and the spatial distribution of collecting localities within sampling units (Table 1, Fig 6, S5 Appendix). On the other hand, the spatial pattern of plant richness across Nicaragua and detectability were the same as in experiments A and B.

Finally, experiment D preserved all four aspects of the data on specimens from Nicaragua (Table 1).

### Analysis

For each experiment, we calculated *S*_*n*.*a*_, *E*[*S*_*n*.*r*_] and *E*[*S*_*n*.*ser*_] in each sampling unit, for *n* = 200 and *n* = 500. To calculate *S*_*n*.*a*_ we chronologically sorted specimens in each sampling unit, using data on collection date. Then, we determined the number of species in the first *n* specimens. We calculated *E*[*S*_*n*.*r*_] by taking 1,000 random samples, each of *n* specimen records, and calculating mean number of species across these samples. To obtain *E*[*S*_*n*.*ser*_] we took 1,000 spatially explicit samples of *n* specimens in each sampling unit, and calculated the mean number of species across these samples. The initial specimen in each of these spatially explicit samples was selected randomly among the specimens in the sampling unit. Subsequent specimens were added to the sample according to their geographic distance to the initial specimen. Specimens closer to the initial specimen were added first, until *n* specimens were included in the sample.

To test prediction 1, we measured the area covered by subsets of *n* specimens, obtained through individual-based and spatially explicit rarefaction, as the mean (across rarefaction subsets) of the number of 1 x 1 km grid cells with at least one specimen included in the subset of *n* specimens. According to prediction 1, the variance in mean area covered by subsets of *n* specimens should be lower for spatially explicit than for individual-based rarefaction. We therefore considered that prediction 1 was empirically supported if the observed Pitman Morgan test statistic [39] differed in the predicted direction from a null distribution generated by permutation using a significance level α = 0.05. We adopted this permutation approach due to the sensitivity of the Pitman Morgan equal variance test to non-normal distribution of dependent samples [39]. The data, paired by sampling unit, was permuted 1,000 times by randomly assigning treatments (i.e. spatially explicit or individual-based rarefaction) to the two values for each sampling unit.

Predictions 2, 3, and 4 were tested using paired-sample Student *t* tests [40]. These predictions focus on differences in values of species richness obtained in different ways, and state that on average across sampling units *E*[*S*_*n*.*r*_] – *S*_*n*.*a*_ > 0, *E*[*S*_*n*.*r*_] – *E*[*S*_*n*.*ser*_] > 0, and | *E*[*S*_*n*.*r*_] – *S*_*n*.*a*_ |–| *E*[*S*_*n*.*ser*_] – *S*_*n*.*a*_ | > 0, respectively. Accordingly, we considered that these predictions were empirically supported if the respective differences in the values of species richness were in the predicted direction, at a statistical significance level α = 0.05.

Prediction 5 was tested using a linear mixed effects model. This prediction focuses on the effect of sampling unit size on the magnitude of overestimation of *E*[*S*_*n*_] incurred by individual-based rarefaction. We used the identity of 100 x 100 km sampling units as random effects, given that some 5 x 5 and 50 x 50 km sampling units were nested within 100 x 100 km sampling units. On the other hand, fixed effects included sampling unit size, a dummy variable coding whether rarefaction was based on subsets of *n* = 200 or *n* = 500, and the interaction between these two variables (Table A in S6 and S7 Appendices for model specification details). We excluded interaction terms when not significant according to a log likelihood ratio test (Tables B in S6 and S7 Appendices). We ran separate analyses for three response variables: *E*[*S*_*n*.*r*_]–*S*_*n*.*a*_, *E*[*S*_*n*.*r*_]–*E*[*S*_*n*.*ser*_], and | *E*[*S*_*n*.*r*_]–*S*_*n*.*a*_,|–| *E*[*S*_*n*.*ser*_]–*S*_*n*.*a*_ |). We considered that prediction 5 was empirically supported if the effect of sampling unit size was significantly positive in each of these three analyses, at a significance level α = 0.05.

All simulation experiments and statistical analyses were implemented in R version 3.3.1 [41]. Simulations of the spatial pattern of biodiversity were performed using packages *MASS* [42], *sp* [43], *rgdal* [44], and *raster* [45]. Additionally, we also used packages *raster* [45], *mapmisc* [46] and *scales* [47] for producing maps, package *nlme* [48] for mixed-effects linear models, and package *PairedData* [49] for the Pitman Morgan test of equal variances.

## Results

### Experiment A

**Fig 7:**
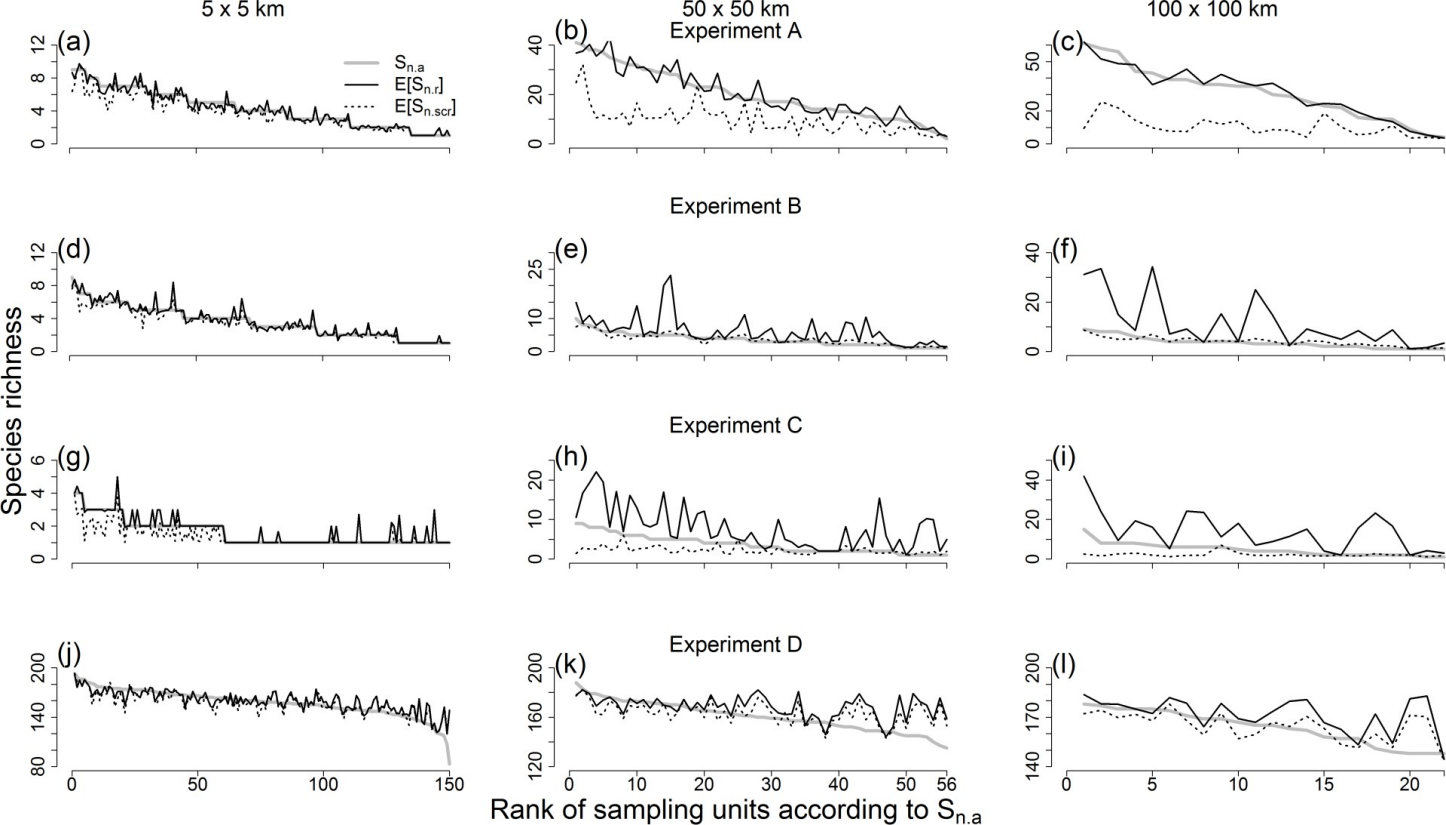
Species richness estimates from accumulation and rarefaction curves at *n* = 200 specimens. Species richness values calculated from accumulation curves (*S*_*n*.*a*_; grey solid line), individual-based rarefaction (*E*[*S*_*n*.*r*_]; black solid line), and spatially explicit rarefaction (*E*[*S*_*n*.*ser*_]; black dotted line) are shown for all sampling units (at three spatial scales: 5 x 5 km, 50 x 50 km, and 100 x 100 km) included in four computer simulation experiments (A, B, C and D).

**Fig 8:**
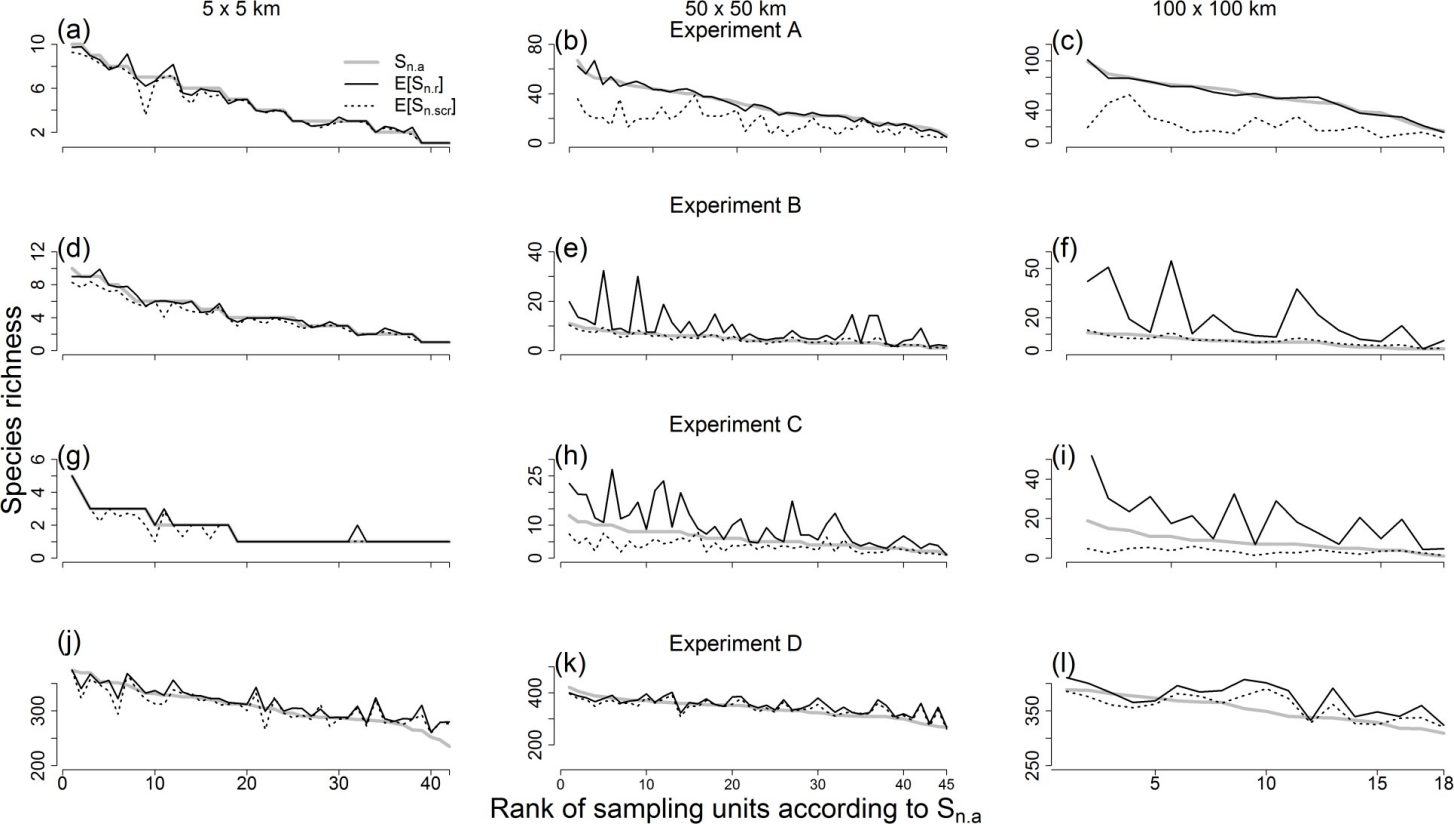
Species richness estimates from accumulation and rarefaction curves at *n* = 500 specimens. Species richness values calculated from accumulation curves (*S*_*n*.*a*_; grey solid line), individual-based rarefaction (*E*[*S*_*n*.*r*_]; black solid line), and spatially explicit rarefaction (*E*[*S*_*n*.*ser*_]; black dotted line) are shown for sampling units (at three spatial scales: 5 x 5 km, 50 x 50 km, and 100 x 100 km) included in four computer simulation experiments (A, B, C and D).

**Fig 9:**
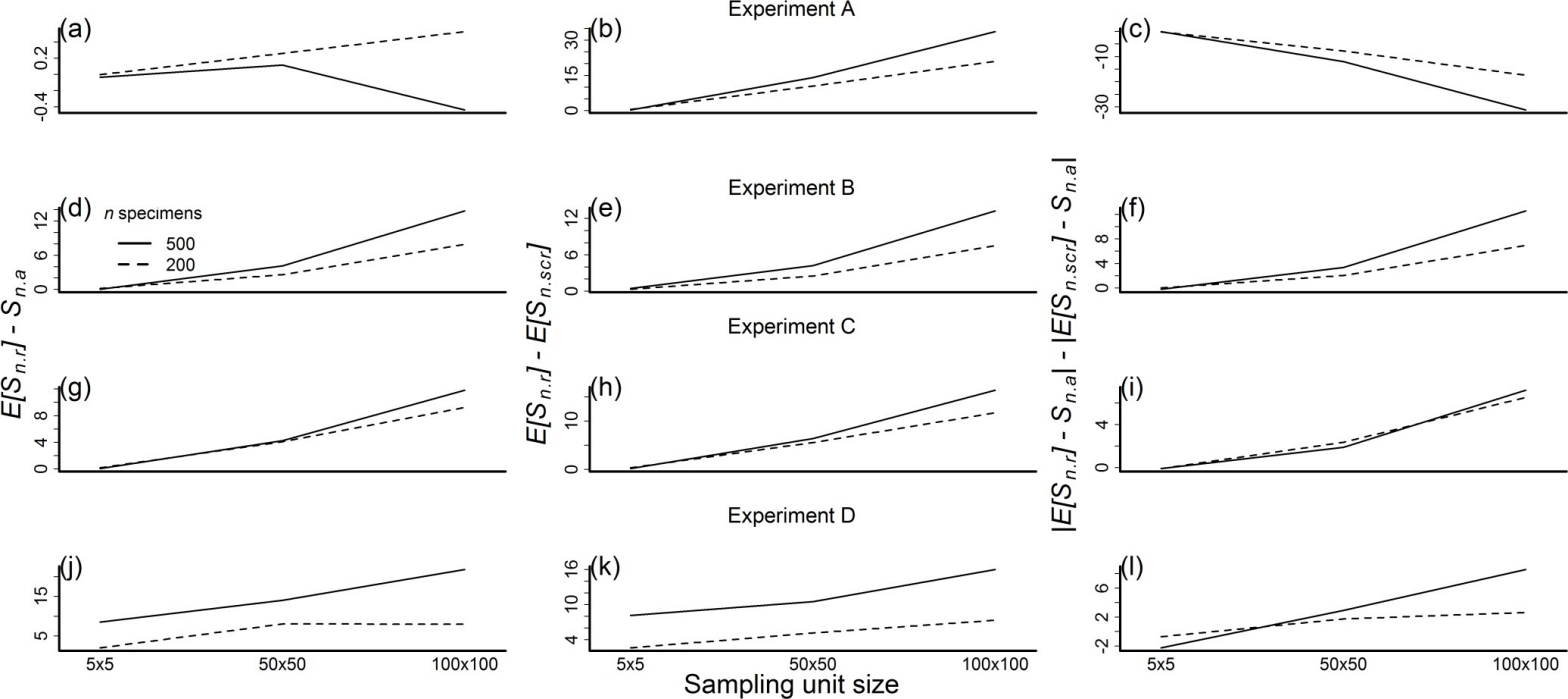
Test of prediction 5. Prediction 5 states that as sampling unit size increases, individual-based rarefaction will more severely overestimate *E*[*S*_*n*_], such that the differences between quantities compared in predictions 2, 3, and 4 should increase with sampling unit size. Differences in quantities from prediction 2, 3 and 4 are shown in the first, second and third columns, respectively. Each row corresponds to an experiment. Solid and dashed lines show results for *n* = 200 and *n* = 500, respectively.

This experiment was designed as a negative control in which individual-based rarefaction was ideal to control differences in sampling effort among sampling units. As expected, predictions 1, 2, and 4 were not supported by data from this experiment (Table 2, Figs 7 and 8). Indeed, results for prediction 4 showed that estimates from individual-based rarefaction were always significantly closer to accumulation curves than those of spatially explicit rarefaction (Table 2). On the other hand, there was support for prediction 3. This result was also expected, because richness estimates based on individual-based rarefaction ought to be generally higher than those based on spatially explicit rarefaction, given spatial aggregation of species occurrences (Fig 2). Finally, also as expected, there was no support for prediction 5. Specifically, although the difference between richness estimates from individual-based rarefaction and spatially explicit rarefaction increased with increasing sampling unit size, the former estimates did not increasingly deviate from accumulation curves as sampling unit size increased (Fig 9). Indeed, as sampling unit size increased, individual-based rarefaction outperformed spatially explicit rarefaction by larger margins (Table 3).

**Table 2:** Tests of predictions 1–4 with experiments A (negative control), B, C, and D.

Experiment	Sampling unit size (km)	*n*	Number of sampling units in the analysis	Prediction 1: (Areaσ^*2*^.*r /Areaσ*^*2*^.*ser*)	Prediction 2: (Mean (*E*[*S*_*n*.*r*_] – *S*_*n*.*a*_))	Prediction 3: (Mean (*E*[*S*_*n*.*r*_] – *E*[*S*_*n*.*ser*_]))	Prediction 4: (Mean (| *E*[*S*_*n*.*r*_]–*S*_*n*.*a*_ |–|*E*[*S*_*n*.*ser*_] – *S*_*n*.*a*_|))
**Experiment A (negative control)**	5x5	200	150	0.272	-0.001	0.409***	-0.244***
	5x5	500	42	0.058	-0.036	0.258**	-0.084
	50x50	200	56	0.399	0.259	10.456***	-7.867***
	50x50	500	45	0.35	0.116	14.041***	-12.011***
	100x100	200	22	0.866	0.531	20.918***	-17.437***
	100x100	500	18	0.057	-0.437	33.68***	-31.214***
**Experiment B**	5x5	200	150	6.012***	0.158***	0.308***	-0.003
	5x5	500	42	1.503*	0.021	0.436***	-0.213**
	50x50	200	56	41.733***	2.593***	2.493***	2.008***
	50x50	500	45	37.094***	4.109***	4.204***	3.316***
	100x100	200	22	22.807***	7.88***	7.493***	6.919***
	100x100	500	18	20.185***	13.802***	13.264***	12.581***
**Experiment C**	5x5	200	150	3.136***	0.148***	0.323***	-0.103*
	5x5	500	42	1.404*	0.048	0.156**	-0.102^.^
	50x50	200	56	49.773***	4.083***	5.544***	2.328***
	50x50	500	45	16.601***	4.236***	6.374***	1.854**
	100x100	200	22	206.143***	9.203***	11.699***	6.521***
	100x100	500	18	169.977***	11.828***	16.369***	7.185**
**Experiment D**	5x5	200	150	3.141***	2.035**	2.596***	-0.721^.^
	5x5	500	42	1.402***	8.531***	8.117***	-2.251
	50x50	200	56	49.829***	8.026***	5.14***	1.731*
	50x50	500	45	16.498***	14.006***	10.512***	**2.922^.^**
	100x100	200	22	203.69***	7.99***	7.309***	**2.609^.^**
	100x100	500	18	166.296***	21.817***	16.02***	8.558*

Prediction 1: variance across sampling units in the area occupied by specimens is less for spatially explicit (*Areaσ*^*2*^.*ser*) than for individual-based rarefaction (Area*σ*^*2*^.*r*); Prediction 2: richness estimates from individual-based rarefaction exceed accumulation curves (*E*[*S*_*n*.*r*_] – *S*_*n*.*a*_ > 0); Prediction 3: richness estimates from individual-based rarefaction exceed those from spatially explicit rarefaction (*E*[*S*_*n*.*r*_] – *E*[*S*_*n*.*ser*_] > 0); Prediction 4: estimates from spatially explicit rarefaction are closer to accumulation curves than estimates from individual-based rarefaction (|*E*[*S*_*n*.*r*_] – *S*_*n*.*a*_| – |*E*[*S*_*n*.*ser*_] – *S*_*n*.*a*_| > 0). Bold values indicate support for predictions, while asterisks indicate level of significance: ^.^ for p-value < 0.1

* for p-value ≤ 0.05, and

*** for p-value ≤ 0.001. Note that some significant values are not in bold because results were in the direction opposite to the respective prediction.

**Table 3:** Test of prediction 5.

		*E*[*S*_*n*.*r*_] – *S*_*n*.*a*_		*E*[*S*_*n*.*r*_] – *E*[*S*_*n*.*ser*_]		| *E*[*S*_*n*.*r*_] – *S*_*n*.*a*_ |–|*E*[*S*_*n*.*ser*_] – *S*_*n*.*a*_|	
Experiment	*n*	Intercept	Slope	Intercept	Slope	Intercept	Slope
**A**	200	0.0023	0.00002	0.0023	0.0021***	-0.2469	-0.0018***
**A**	500	-0.0404	NA	-0.0404	0.0031***	-0.0954	-0.0030***
**B**	200	0.1570	0.0008**	0.3062	0.0007**	-0.0049	0.0007**
**B**	500	0.0171	0.0012***	0.2685	0.0011***	-0.2202	0.0011***
**C**	200	0.1486	0.0010***	0.3275	0.0012***	-0.1005	0.0007***
**C**	500	0.0576	NA	0.1677	0.00142**	-0.1122	NA
**D**	200	2.1312	0.0009**	2.0567	0.0006***	-0.3990	0.0004
**D**	500	9.8337	NA	5.3401	0.0011**	-2.0278	0.0011**

Prediction 5 states that as sampling unit size increases, individual-based rarefaction will more severely overestimate richness, relative to spatially explicit rarefaction. Prediction 5 implies that the following quantities increase as sampling unit size increases: the difference between richness estimates from individual-based rarefaction and accumulation curves (*E*[*S*_*n*.*r*_] – *S*_*n*.*a*_), the difference between richness estimates from individual-based and spatially explicit rarefaction (*E*[*S*_*n*.*r*_] – *E*[*S*_*n*.*ser*_], and the difference between absolute deviations of richness estimates from rarefaction and the accumulation curve (|*E*[*S*_*n*.*r*_]–*S*_*n*.*a*_| – |*E*[*S*_*n*.*ser*_] – *S*_*n*.*a*_|). Estimates of intercepts and slopes are based on the model with lowest AIC (S4 Appendix). Bold values indicate support for prediction 5, while asterisks indicate level of significance:

* for p-value < 0.05

** for p-value ≤ 0.01 and

*** for p-value ≤ 0.001. NA indicates that slope value for *n* = 500 did not differ from slope value for *n* = 200. Note that significant values for Experiment A in the column for |*E*[*S*_*n*.*r*_] – *S*_*n*.*a*_| – |*E*[*S*_*n*.*ser*_] – *S*_*n*.*a*_| are not in bold because prediction 5 was not supported, since regression coefficients were in the direction opposite to that predicted.

### Experiments B, C, and D

Experiments B, C, and D consistently supported predictions 1–4, but only for the larger sampling units of 50 x 50 and 100 x 100 km (Table 2). Thus, in these instances, spatially explicit rarefaction was better than individual-based rarefaction to control for differences in sampling effort among sampling units (Figs 7 and 8). In contrast, none of the three experiments showed evidence that spatially explicit rarefaction performed better than individual-based rarefaction at sampling units of 5 x 5 km (Table 2, Figs 7 and 8).

Overall, experiments B, C, and D supported prediction 5, albeit to varying extents. In particular, the difference between richness estimates from individual-based rarefaction and accumulation curves (*E*[*S*_*n*.*r*_]–*S*_*n*.*a*_) increased with sampling unit size in experiments B, C, and D and this increase was higher for *n* = 500 than for *n* = 200 in experiment B (Table 3, Fig 9). The difference between richness estimates from individual-based and spatially explicit rarefaction (*E*[*S*_*n*.*r*_]–*E*[*S*_*n*.*ser*_]) consistently increased with sampling unit size, and more for *n* = 500 than for *n* = 200 in all three experiments (Table 3, Fig 9). Last, the difference between absolute deviations of rarefaction estimates from accumulation curves (|*E*[*S*_*n*.*r*_] – *S*_*n*.*a*_| – |*E*[*S*_*n*.*ser*_] – *S*_*n*.*a*_|) increased with sampling unit size in experiments B and C, with stronger effect for *n* = 500 than for *n* = 200 in experiment B (Table 3, Fig 9). In contrast, this increase was significant only for *n* = 500 in experiment D (Table 3, Fig 9).

## Discussion

Natural history museum specimens often constitute the only available data for estimating broad-scale species richness of highly diverse taxa. However, museum specimens are typically collected without standardized sampling protocols [24]. Consequently, data from museum specimens are likely characterized by multiple biases that may impact estimates of broad-scale patterns of species richness and related analyses [50]. Thus, it is useful to develop approaches to control for bias in these data, such as unequal sampling effort across space, but little previous work addresses this issue. Here we explored the performance of two versions of rarefaction, which is thought to be a promising approach for estimating broad-scale richness with data from museum specimens while accounting for heterogeneous sampling effort across space [8,26]. Specifically, we examined the working hypothesis that, when estimating broad-scale species richness using data from museum specimens, spatially explicit rarefaction [34,35] is less biased than individual-based rarefaction [11], because it reduces overestimation due to the spatial aggregation of sampling activities. From this hypothesis we derived five predictions that can be tested using data from museum specimens. Therefore, these predictions can be used to evaluate the performance of individual-based and spatially explicit rarefaction and, thus, help determine if any of these two approaches may be reasonably applied to a particular study system.

We tested the predictions using four computer simulation experiments. One of these experiments was a negative control (experiment A), designed to meet the assumptions of individual-based rarefaction and thus render the working hypothesis false. In particular, in experiment A, species occurrences were spatially aggregated, but sampling effort was uniformly distributed within sampling units. Consistent with expectations, in this experiment, richness estimates from individual-based rarefaction were not significantly different from accumulation curves and were better than estimates from spatially explicit rarefaction (Table 2, Figs 7 and 8). Thus, experiment A serves as a proof of concept. It shows that, when false, the working hypothesis can be rejected by empirically examining predictions 1–5. This result supports the logic underlying the derivation of predictions, and the idea that the predictions are useful to explore the performance of individual-based and spatially explicit rarefaction as approaches to estimate broad-scale species richness based on data from museum specimens.

The design of three other experiments (B, C, and D) met key assumptions of the working hypothesis: spatial aggregation of species occurrences and sampling effort within sampling units. Yet results from these experiments supported the working hypothesis only in analyses of the two larger sampling units (50 x 50 and 100 x 100 km, Table 2). Indeed, analysis of the smaller sampling units (5 x 5 km) suggested that individual-based rarefaction outperformed spatially explicit rarefaction in several instances (prediction 4 in Table 2). There seem to be two primary explanations for lack of support for the working hypothesis in analysis of the smallest sampling units. First, the ratio of the variances in the area occupied by subsets of specimens drawn by rarefaction (prediction 1 in Table 2) was markedly smaller for 5 x 5 km than for 50 x 50 and 100 x 100 km sampling units. This suggests that weaker spatial aggregation of sampling effort at smaller sampling units led to lack of support for the working hypothesis. Second, spatial heterogeneity in species composition within sampling units may have been low in small sampling units relative to large sampling units. If sampling units are relatively homogenous in species composition, individual-based and spatially explicit rarefaction may yield similar estimates of species richness, even when sampling effort is spatially aggregated (S8 Appendix, see also [33]). Thus, under these two potential explanations, spatially explicit rarefaction would increasingly outperform individual-based rarefaction as sampling unit size increases. This is consistent with the results supporting prediction 5 (Table 3, Fig 9). Further studies that test the predictions developed here may help establish the generality of these findings.

**Fig 10:**
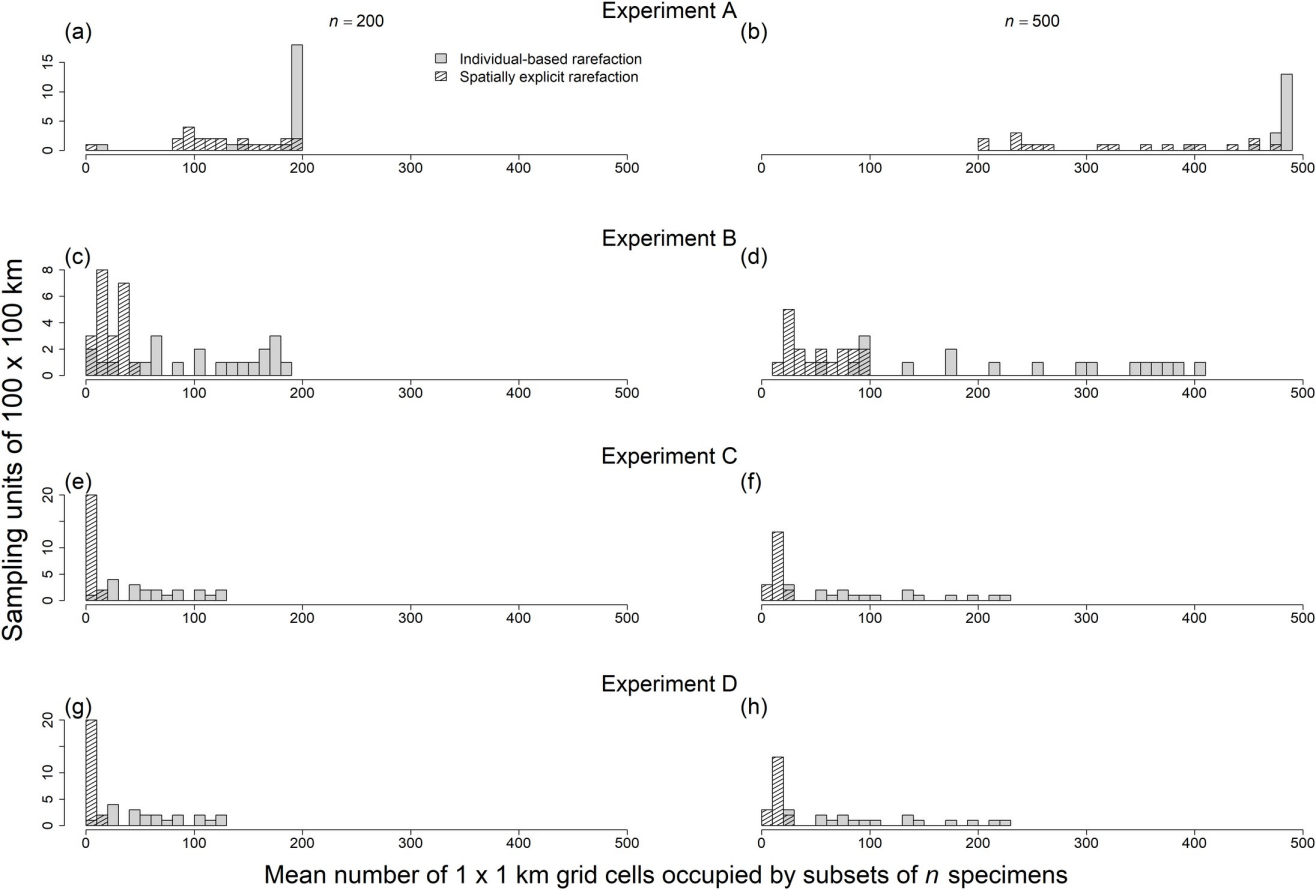
Spatially explicit rarefaction reduced variation among sampling units in area occupied by subsets of *n* specimens. When sampling was uniformly distributed within sampling units (experiment A), spatially explicit rarefaction did not reduce variation among sampling units in the area occupied by subsets of *n* specimens (a)–(b). However, when sampling activities were spatially aggregated (experiment B, C and D), spatially explicit rarefaction did reduce variation among sampling units in the area occupied by subsets of *n* specimens (c)–(h). Here only 100 x 100 km sampling units are shown, but results were similar for sampling units of 50 x 50 km and, to a lesser extent, 5 x 5 km (prediction 1 in Table 2). Area occupied by subsets of *n* specimens is defined as the number of 1 x 1 km grid cells occupied within a sampling unit.

The fundamental difference between spatially explicit and individual-based rarefaction is that the former incorporates spatial information about the collection localities of specimens, and aims to control for variation in the spatial extent of collection localities among sampling units. In particular, spatially explicit rarefaction attempts to reduce variation among sampling units in the area occupied by *n* specimens drawn from the pool of *N* specimens. This was effectively accomplished in experiments B, C and D (prediction 1 in Table 2, Fig 10). Nonetheless, it is worth asking if different implementations of spatially explicit rarefaction may accomplish this goal better. Our implementation of spatially explicit rarefaction was based on the *k*-nearest neighbor distance method: an initial specimen was selected randomly among the specimens in the sampling unit, and subsequent specimens were added to the sample according to their geographic distance to the initial specimen. There are other methods to draw spatially explicit subsets of *n* specimens from the pool of *N* specimens collected in a sampling unit. These methods may be distance-, graph-, or grid-based including *k*-nearest centroid neighbor, natural neighbors defined through Voronoi tessellation, and γ-neighbors [34,51]. It would be instructive to examine the extent to which these other methods may improve the performance of spatially explicit rarefaction as applied to museum data.

Estimates of *E*[*S*_*n*_] based on museum specimen data may only be approximations with potentially large measurement error, because these data are not commonly obtained using standardized sampling techniques, which is an assumption of rarefaction [18]. Yet, in principle, measurement error may be appropriately subsumed in the error terms of models relating *E*[*S*_*n*_] to variables hypothesized to determine spatial richness patterns [21,52]. Moreover, if we understand the structure of measurement error associated with estimates of *E*[*S*_*n*_] based on museum specimens, it may be possible to develop ways to reduce this measurement error. Here we took a step in this direction, by examining measurement error introduced by spatial aggregation of sampling effort. Further work on this and other sources of measurement error [24,25], may increase our ability to estimate broad-scale spatial patterns of richness for a wide range of taxa. In turn, this would increase the testability of major ecological and evolutionary hypotheses, and allow prioritizing areas for biodiversity conservation with increased input from data on poorly known taxa.

## Supporting information

S1 Appendix Differences in spatial distribution of sampling effort confound species richness comparisons between sampling units. (DOCX)

S2 Appendix Specimen records downloaded in April 2015 from Tropicos database under the Flora de Nicaragua Project. (TXT)

S3 Appendix Simulation of the spatial pattern of plant richness across Nicaragua. (DOCX)

S4 Appendix R code and data files for simulating the spatial pattern of plant richness across Nicaragua. (ZIP)

S5 Appendix R code and data files for simulating experiments A, B, and C.
(ZIP)

S6 Appendix Specification of mixed effects models used to test prediction 5. (DOCX)

S7 Appendix AIC_c_ values for mixed effects model used to test prediction 5. (DOCX)

S8 Appendix Rarefaction and accumulation curves for sampling units with low and high spatial heterogeneity in species composition. (DOCX)

S9 Appendix Distribution of number of specimens per sampling unit at three spatial scales (5 x 5, 50 x 50, and 100 x 100 km). (DOCX)
