# Supplementary material for: Using museum specimens to estimate broad-scale species richness: Exploring the performance of individual-based and spatially explicit rarefaction
Source: PLoS One. 2018 Oct 31;13(10):e0204484. doi: 10.1371/journal.pone.0204484 (PMC6209151; doi:10.1371/journal.pone.0204484)

**S1 Appendix. Differences in the pattern of spatial aggregation of collection localities confound comparisons of species richness between sampling units. Spatial aggregation of specimens is higher in (a) and (b) than in (c). This difference in spatial aggregation of specimens between (b) and (c) confounds the comparison of accumulation curves in (e) and (f) even when sampling effort is the same.**


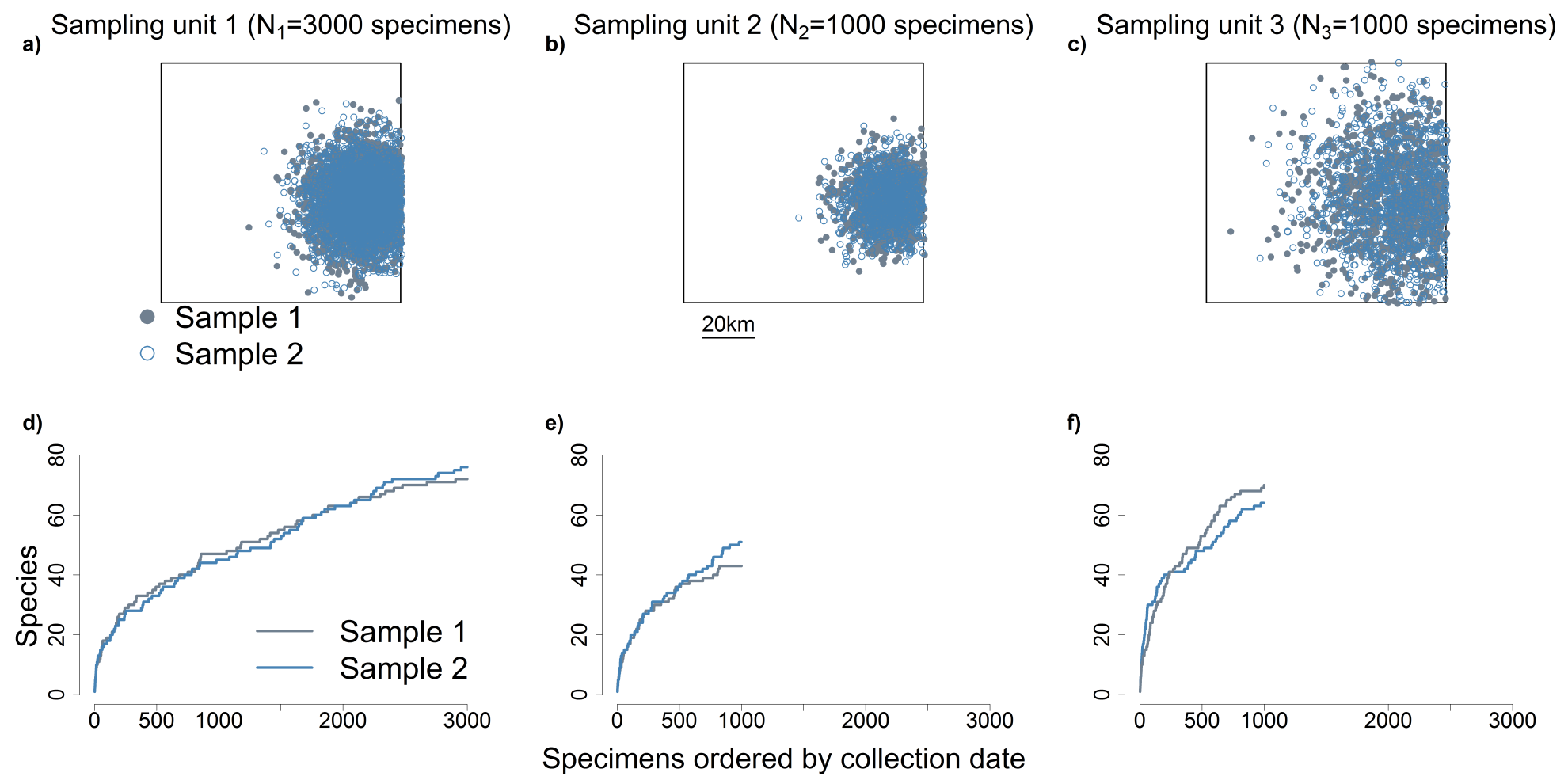

Supplement: S1 Appendix — (DOCX) [file pone.0204484.s001.docx]
