# Supplementary material for: Using museum specimens to estimate broad-scale species richness: Exploring the performance of individual-based and spatially explicit rarefaction
Source: PLoS One. 2018 Oct 31;13(10):e0204484. doi: 10.1371/journal.pone.0204484 (PMC6209151; doi:10.1371/journal.pone.0204484)
Supplement: S3 Appendix — (DOCX) [file pone.0204484.s003.docx]

**S3 Appendix.** Simulation of the spatial pattern of plant richness across Nicaragua.

We simulated the spatial pattern of plant richness across Nicaragua (second column in Table 1) using a model based on continuum theory (McGill & Collins 2003; McGill 2010). This continuum theory model has been shown to simulate communities with realistically shaped species abundance distributions and species area curves (McGill 2010). According to this model, the abundance of species *i* at a given site with geographical coordinates *X* and *Y* is:

$N_{i} \left( X,Y \right)=\frac{{NMAX}_{i}\times\exp\left( {|X- \mu_{i1}|}^{2} \right)+ {NMAX}_{i}\times\exp\left( {|Y- \mu_{i2}|}^{2} \right)}{{2\sigma}_{i}^{2}}$ (equation 1),

where ${NMAX}_{i}$ is the maximum abundance of species *i* across its whole geographic range, *µ_i1_* and *µ_i2_* are the geographical coordinates for the site where this maximum abundance is reached, and $\sigma_{i}$ is a geographic scaling constant for range size. When $N_{i} \left( X, Y \right)$< 1, we transformed $N_{i} \left( X, Y \right)$ to zero.

We simulated the geographic range of species by randomly sampling *µ_i1_* and *µ_i2_* coordinates from a uniform distribution across 1 x 1 km grid cells covering the Neotropics (not just Nicaragua), while *NMAX_i_ and* $\sigma_{i}$ were sampled from log normal distributions with means 18 and 5, and standard deviations 5 and 4, respectively. Using equation 1, we simulated species across the Neotropics until we obtained 15,000 species with geographic ranges that overlapped Nicaragua, at least partially. Abundance of each of these 15,000 species across Nicaragua was simulated at a resolution of 1 x 1 km grid cells. Therefore, we obtained the total number and identity of simulated species occurring in each 1 x 1 km grid cell across Nicaragua, and the respective species abundance distribution (SAD, Fig 1a).
