# Supplementary material for: Using museum specimens to estimate broad-scale species richness: Exploring the performance of individual-based and spatially explicit rarefaction
Source: PLoS One. 2018 Oct 31;13(10):e0204484. doi: 10.1371/journal.pone.0204484 (PMC6209151; doi:10.1371/journal.pone.0204484)
Supplement: S6 Appendix — (DOCX) [file pone.0204484.s006.docx]

**S6 Appendix. Specification of mixed effects models used to test prediction 5.**

a. Structure of random effects. b. Structure of fixed effects. The random effects refer to the identity of 100 x 100 km sampling units within which each 5 x 5 and 50 x 50 km sampling unit was nested.

1. Random effects structure

| **Model** | **Structure of random effects** |
| --- | --- |
| **A** | random intercept (1\|Sampling_unit_100x100) |
| **B** | random slope 1 (Sampling unit size\| Sampling_unit_100x100) |
| **C** | random slope 2 (*n*\| Sampling_unit_100x100) |
| **D** | random intercept and slope 1 (1 + Sampling unit size\| Sampling_unit_100x100) |
| **E** | random intercept and slope 2 (1 + *n*\| Sampling_unit_100x100) |
| **F** | random intercept and slopes 1 & 2  (1 + Sampling unit size + *n*\| Sampling_unit_100x100) |

1. Fixed effects structure

| **Model** | **Structure of fixed effects** |
| --- | --- |
| **Complete** | Response variable = intercept + Sampling unit size +  *n* + Sampling unit size * *n* |
| **No interaction** | Response variable = intercept + Sampling unit size +  *n* |
