# Supplementary material for: Using museum specimens to estimate broad-scale species richness: Exploring the performance of individual-based and spatially explicit rarefaction
Source: PLoS One. 2018 Oct 31;13(10):e0204484. doi: 10.1371/journal.pone.0204484 (PMC6209151; doi:10.1371/journal.pone.0204484)
Supplement: S7 Appendix — (DOCX) [file pone.0204484.s007.docx]

**S7 Appendix.** AIC_c_ values for mixed effects model used to test prediction 5.

The structure of these models is described in S2 Appendix. a. The random structure used to test prediction 5 was the one with the lowest AIC_c_. b. The fixed structure used to test prediction 5 was also that with lowest AIC_c_. Additionally, we examined caterpillar plots to confirm whether random intercepts and/or slopes were necessary in the mixed model. We conducted model diagnostics on the level-1 residuals to check the validity of model assumptions for mixed models. The lowest AIC_c_ values, corresponding to selected models, are shown in bold font. When there was a tie in AIC_c_ values, we selected the model with simplest random structure.

1. AIC_c_ values for different structures of random effects

| **Experiment** | **Response variable** | **Model A** | **Model B** | **Model C** | **Model D** | **Model E** | **Model F** |
| --- | --- | --- | --- | --- | --- | --- | --- |
| **A** | $E\left[ S_{n.r} \right]$ – $S_{n.a}$ | 1454.295 | **1422.882** | 1458.353 | 1426.5 | 1458.353 | 1433.015 |
| **A** | $E\left[ S_{n.r} \right]$ – $S_{n.ser}$ | 2292.058 | **2098.938** | 2281.484 | 2100.528 | 2281.484 | 2100.571 |
| **A** | \|$E\left[ S_{n.r} \right]$ – $S_{n.a}$ \| – \|$E\left[ S_{n.ser} \right]$ – $S_{n.a}$\| | 2246.035 | **2060.766** | 2235.642 | 2064.693 | 2235.642 | 2076.05 |
| **B** | $E\left[ S_{n.r} \right]$ – $S_{n.a}$ | 1942.681 | **1593.839** | 1919.6 | 1597.713 | 1919.6 | 1596.362 |
| **B** | $E\left[ S_{n.r} \right]$ – $S_{n.ser}$ | 1913.51 | 1554.358 | 1893.251 | 1557.585 | 1893.251 | **1551.474** |
| **B** | \|$E\left[ S_{n.r} \right]$ – $S_{n.a}$ \| – \|$E\left[ S_{n.ser} \right]$ – $S_{n.a}$\| | 1919.655 | **1576.473** | 1897.101 | 1580.435 | 1897.101 | 1579.414 |
| **C** | $E\left[ S_{n.r} \right]$ – $S_{n.a}$ | 1821.213 | **1555.679** | 1815.534 | 1559.67 | 1815.534 | 1571.624 |
| **C** | $E\left[ S_{n.r} \right]$ – $S_{n.ser}$ | 1965.321 | **1641.081** | 1957.395 | 1643.811 | 1957.395 | 1653.355 |
| **C** | \|$E\left[ S_{n.r} \right]$ – $S_{n.a}$ \| – \|$E\left[ S_{n.ser} \right]$ – $S_{n.a}$\| | 1756.086 | **1587.821** | 1754.522 | 1589.571 | 1754.522 | 1603.344 |
| **D** | $E\left[ S_{n.r} \right]$ – $S_{n.a}$ | 2737.919 | **2733.404** | 2735.202 | 2733.404 | 2735.202 | 2735.881 |
| **D** | $E\left[ S_{n.r} \right]$ – $S_{n.ser}$ | 2080.688 | 2081.497 | **2062.947** | 2081.497 | 2062.947 | 2065.327 |
| **D** | \|$E\left[ S_{n.r} \right]$ – $S_{n.a}$ \| – \|$E\left[ S_{n.ser} \right]$ – $S_{n.a}$\| | 2300.176 | 2289.445 | 2293.198 | 2289.445 | 2287.98 | **2284.09** |

1. AIC_c_ values for different structures of fixed effects. Asterisks show significant differences according to the log likelihood ratio test: * for p-value ≤ 0.05, ** for p-value ≤ 0.01, and *** for p-value ≤ 0.001.

|  | $\boldsymbol{E}\left[ \boldsymbol{S}_{\boldsymbol{n.r}} \right]$ **–** $\boldsymbol{S}_{\boldsymbol{n.a}}$ | |  | $\boldsymbol{E}\left[ \boldsymbol{S}_{\boldsymbol{n.r}} \right]$ **–** $\boldsymbol{E[S}_{\boldsymbol{n.ser}}\boldsymbol{]}$ | |  | **\|**$\boldsymbol{E}\left[ \boldsymbol{S}_{\boldsymbol{n.r}} \right]$ **–** $\boldsymbol{S}_{\boldsymbol{n.a}}$ **\| – \|**$\boldsymbol{E}\left[ \boldsymbol{S}_{\boldsymbol{n.ser}} \right]$ **–** $\boldsymbol{S}_{\boldsymbol{n.a}}$**\|** | |
| --- | --- | --- | --- | --- | --- | --- | --- | --- |
| **Experiment** | **Complete** | **No interaction** |  | **Complete** | **No interaction** |  | **Complete** | **No interaction** |
| **A** | 1457.840 | 1455.398 |  | **2259.480***** | 2278.092 |  | **2229.705***** | 2255.983 |
| **B** | **1943.071***** | 1953.683 |  | **1914.596***** | 1924.369 |  | **1921.706***** | 1932.563 |
| **C** | 1810.905 | 1811.236 |  | **1947.011*** | 1951.231 |  | 1757.121 | 1754.515 |
| **D** | 2739.475 | 2737.919 |  | 2080.688 | 2080.489 |  | **2300.176**** | 2304.942 |
