# Supplementary material for: Using museum specimens to estimate broad-scale species richness: Exploring the performance of individual-based and spatially explicit rarefaction
Source: PLoS One. 2018 Oct 31;13(10):e0204484. doi: 10.1371/journal.pone.0204484 (PMC6209151; doi:10.1371/journal.pone.0204484)

**S8 Appendix. Rarefaction and accumulation curves for sampling units with low and high spatial heterogeneity in species composition.**

Two contrasting levels of spatial heterogeneity in species composition are shown: low spatial heterogeneity in species composition in the first column, and high spatial heterogeneity in species composition in the second column. The top row shows heterogeneity in species composition among 1 x 1 km grid cells within the sampling unit, measured as Bray-Curtis dissimilarity. Accumulation curves (gray) as well as individual-based (slate grey) and spatially explicit (black) rarefaction curves are shown for a 100 x 100 km sampling unit where 2,000 virtual species occur, and where 17,473 specimens have been collected. The distribution of collection localities within the sampling unit was simulated as in Experiment B, based on an “expanding” bivariate normal distribution (see text and Table 1). Contrasting levels of spatial heterogeneity were simulated using a model based on continuum theory (McGill & Collins, 2003, Appendix S1). In both cases (i.e., low and high spatial heterogeneity in species composition), the log normal distribution from which *NMAX_i_ values* were sampled had mean 4 and standard deviation 2. On the other hand, the log normal distribution from which $\sigma_{i}$ values were sampled had standard deviation 4 in both cases, but different means. The mean for low spatial heterogeneity was 25, and for high spatial heterogeneity was 5. Thus, species were more narrowly distributed in the high than in the low heterogeneity case. Red dotted lines indicate the size of subset of *n* specimens, *n* = 200 and *n* = 500. For all rarefaction curves, 95 percentile envelopes are shown in light gray.


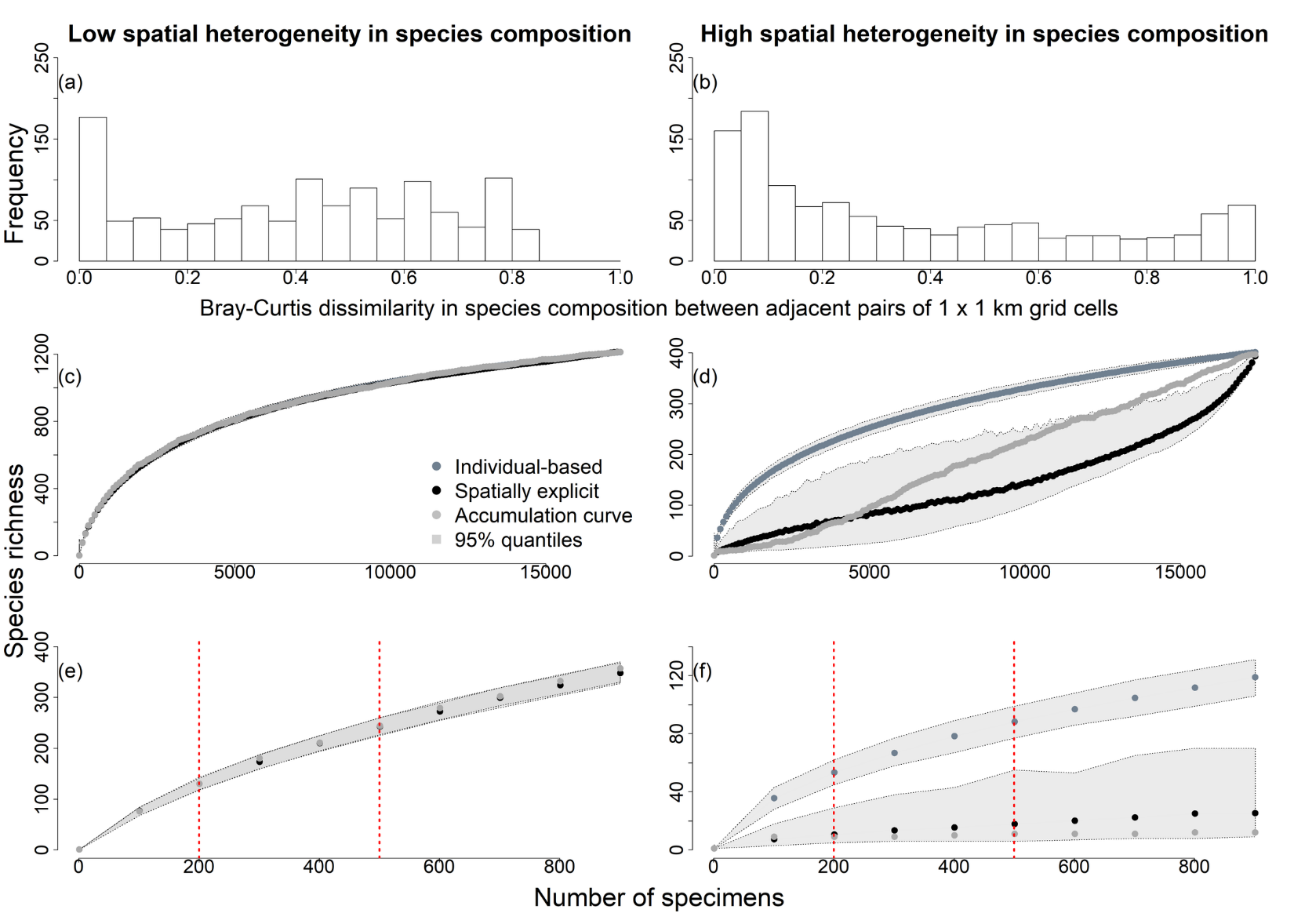

Supplement: S8 Appendix — (DOCX) [file pone.0204484.s008.docx]
