# Supplementary material for: Using museum specimens to estimate broad-scale species richness: Exploring the performance of individual-based and spatially explicit rarefaction
Source: PLoS One. 2018 Oct 31;13(10):e0204484. doi: 10.1371/journal.pone.0204484 (PMC6209151; doi:10.1371/journal.pone.0204484)

**S9 Appendix.** Distribution of number of specimens per sampling unit at three spatial scales (5 x 5, 50 x 50, and 100 x 100 km).


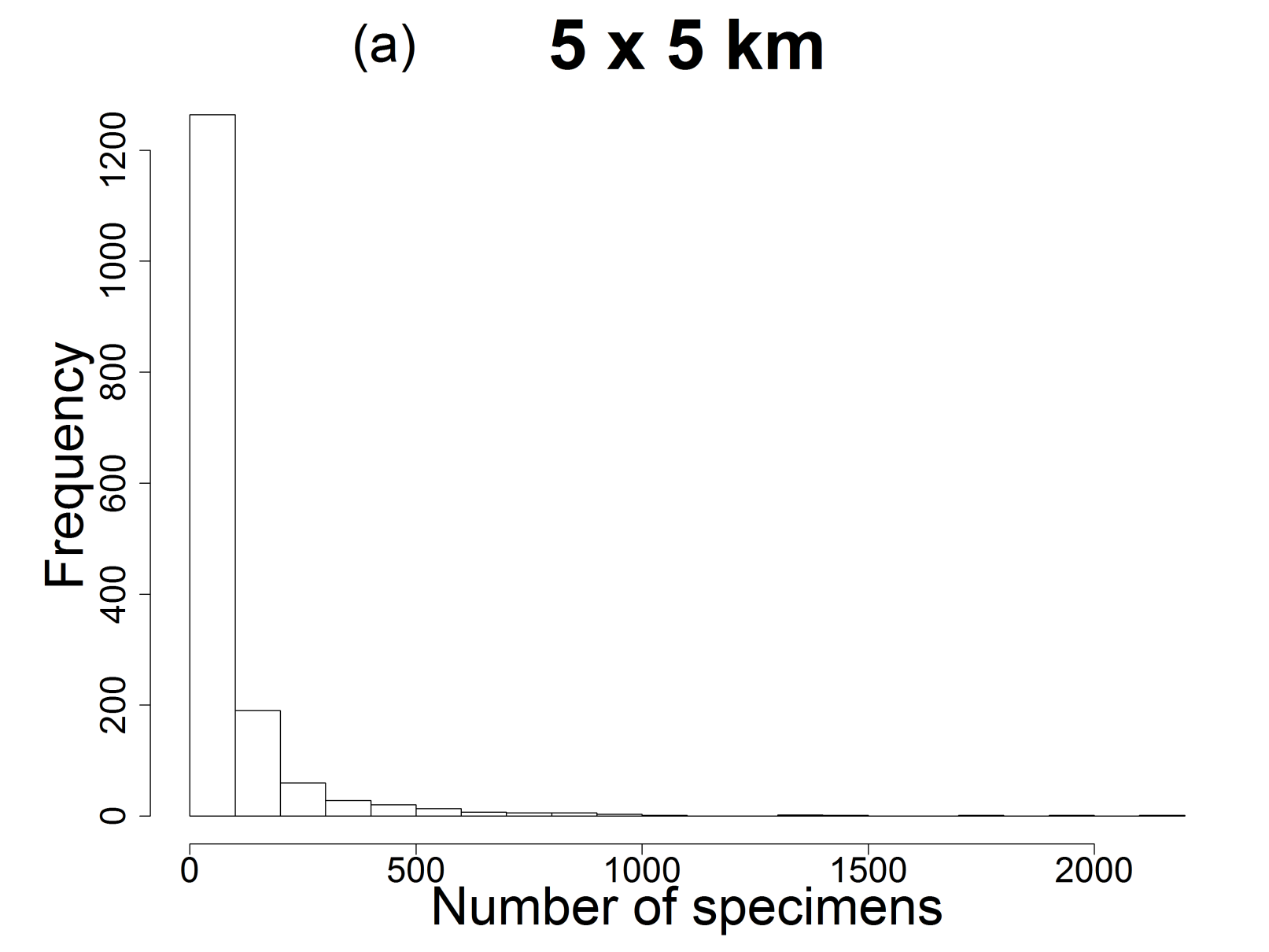


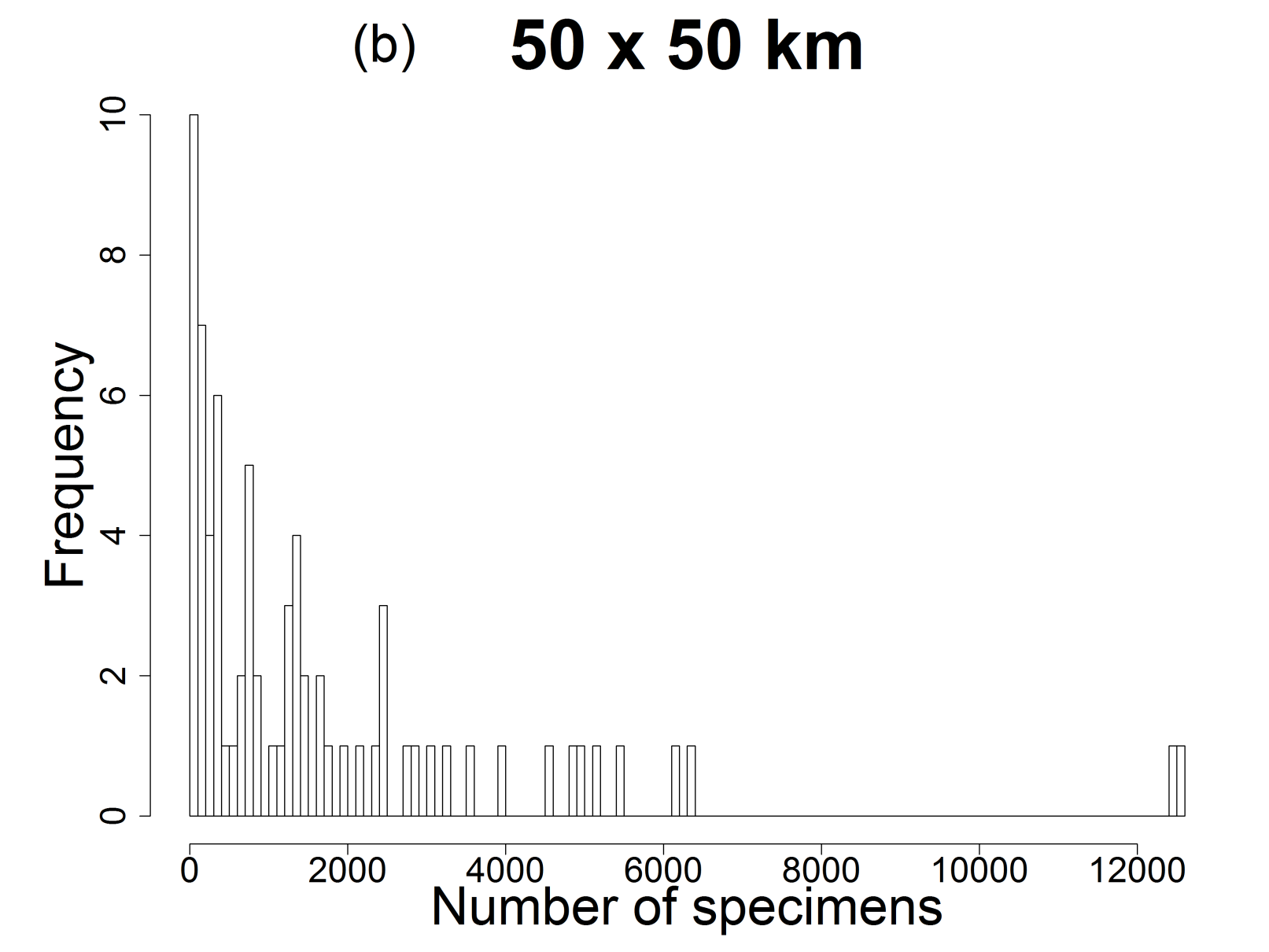


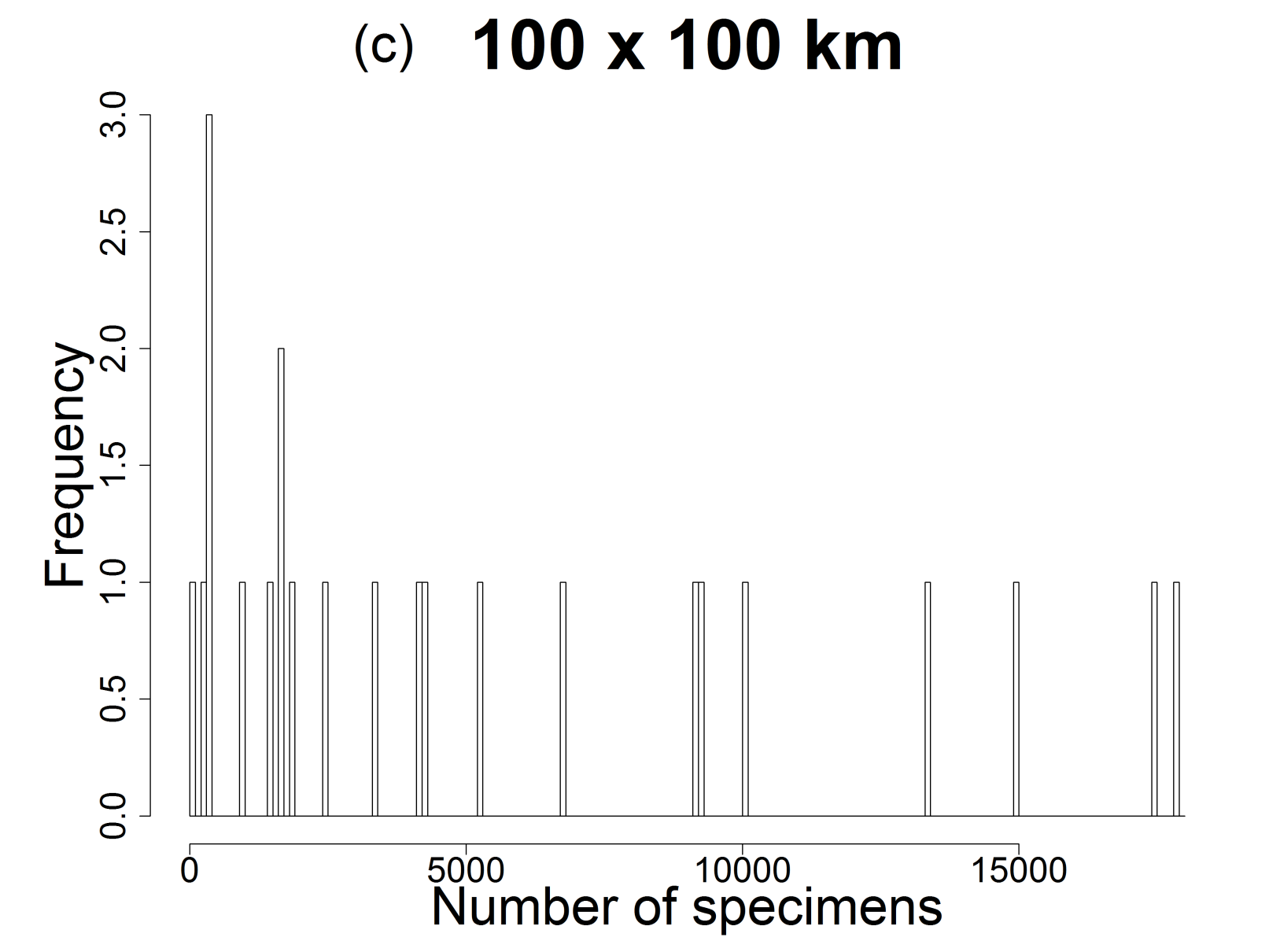

Supplement: S9 Appendix — (DOCX) [file pone.0204484.s009.docx]
